# Down-regulated cylindromatosis enhances NF-κB activation and aggravates inflammation in HBV-ACLF patients

**DOI:** 10.1080/22221751.2022.2077128

**Published:** 2022-06-07

**Authors:** Xueyun Zhang, Yao Zhang, Pu Zhou, Jingwen Ai, Xiaoqin Liu, Quanbao Zhang, Zhengxin Wang, Hongyan Wang, Wenhong Zhang, Jiming Zhang, Yuxian Huang

**Affiliations:** a Department of Infectious Diseases, Shanghai Key Laboratory of Infectious Diseases and Biosafety Emergency Response, Shanghai Institute of Infectious Diseases and Biosecurity, National Medical Center for Infectious Diseases, Huashan Hospital, Fudan University, Shanghai, People's Republic of China; b Huashan Worldwide Medical Center, Huashan Hospital, Fudan University, Shanghai, People’s Republic of China; c Department of General Surgery, Huashan Hospital, Fudan University, Shanghai, People’s Republic of China; d State Key Laboratory of Cell Biology, Shanghai Institute of Biochemistry and Cell Biology, Center for Excellence in Molecular Cell Science, Chinese Academy of Sciences, University of Chinese Academy of Sciences, Shanghai, People’s Republic of China; e Key Laboratory of Medical Molecular Virology (MOE/MOH), Shanghai Medical College, Fudan University, Shanghai, People’s Republic of China; f Department of Infectious Diseases Jing’An Branch of Huashan Hospital, Fudan University, Shanghai, People’s Republic of China; g Department of Hepatology, Shanghai Public Health Clinical Center, Fudan University, Shanghai, People's Republic of China

**Keywords:** RNA-sequencing, acute-on-Chronic liver failure, CYLD, NF-κB, chemotaxis

## Abstract

The pathogenesis of liver in patients with hepatitis B virus-associated acute chronic liver failure (HBV-ACLF) remains largely unknown. We aimed to elucidate the molecular mechanism underlying the pathogenesis of liver in HBV-ACLF patients by using multiple approaches including transcriptome analysis. We performed transcriptomic sequencing analysis on the liver of HBV-ACLF patients (*n* = 6), chronic hepatitis B (*n* = 6), liver cirrhosis (*n* = 6) and normal control (*n* = 5), then explored the potential pathogenesis mechanism in liver specimen from another 48 subjects and further validated the molecular and cellular mechanisms using THP-1 cells. RNA-sequencing data analysis indicated that, among the genes up-regulated in HBV-ACLF, genes related to inflammatory response and chemotaxis accounted for a large proportion of the total DEGs. A number of key chemokines (CCL2, CCL5, CCL20, CXCL5, CXCL6, CXCL8) and NF-ĸB pathway were identified to be robust in the liver samples from HBV-ACLF patients. Interestingly, cylindromatosis (CYLD) was found to be downregulated in the liver of HBV-ACLF patients, in line with the well-established role of CYLD in regulating most of the chemokines and pro-inflammatory cytokines (CCL2, CCL5, CCL20, CXCL5, CXCL6, CXCL8, IL-6, IL-1β) via inhibition of NF-ĸB. Indeed, the knockdown of CYLD resulted in sustained activation of NF-ĸB in macrophages and enhanced chemokines and inflammatory cytokines production, which in turn enhanced chemotactic migration of neutrophil, monocyte, T lymphocytes, and NK cell. In conclusions, down-regulated CYLD aggravated inflammatory cell chemotaxis through enhancing NF-κB activation in HBV-ACLF patients, thereby participating in the pathogenesis of HBV-ACLF injury.

## Introduction

Acute-on-chronic liver failure is a syndrome characterized by acute liver function deterioration in patients with pre-existing chronic liver disease, exhibiting liver and extrahepatic organ failure and a high short-term mortality [1]. To date, more than 250 million individuals are chronically infected with Hepatitis B virus (HBV) worldwide [2]. Hepatitis B virus-related ACLF (HBV-ACLF), which caused high mortality in the Asia-Pacific and African regions, exhibits specific clinical characteristics. These clinical features includes higher prevalence of liver failure and relatively lower prevalence of kidney failure, making HBV-ACLF highly distinguishable from the alcoholic liver disease-related ACLF in western populations [3].

Currently there is no effective therapeutic agent available for treating ACLF, partially due to lack of full understanding of its pathogenesis. To date, most studies have focused on pathogenesis studies using peripheral blood samples, including secondary infection caused by immune paralysis, inflammatory damage mediated by Th17 cell, and mitochondrial dysfunction in leukocyte [4–8]. However, the direct molecular and cellular contributing factors in the liver might play a crucial role in liver and extrahepatic organ failure, which, however, has not been fully studied. Liver histopathology analysis of HBV-ACLF patients revealed extensive necrosis along the terminal hepatic veins and spanning multiple adjacent cirrhotic nodules, with varying degrees of liver progenitor cell-derived regeneration, cholestasis, and ductular bilirubinostasis [9].

To further elucidate the molecular pathogenesis of the liver in HBV-ACLF patients, we carried out RNA-seq analysis on the liver tissues from HBV-ACLF, chronic hepatitis B, liver cirrhosis, and normal control patients. Here, our liver transcriptome sequencing data analysis indicated that the chemotaxis of inflammatory cells may contribute significantly to the pathogenesis of HBV-ACLF. Various chemokines were significantly elevated in the liver of HBV-ACLF patients and most of them were the target genes of NF-ĸB [10–12]. The NF-ĸB signalling pathway has been known to play a key role in regulating inflammation, immune response, oncogenesis, and apoptosis [13]. Moreover, transcriptome sequencing data analysis also showed that the activation of NF-κB in the liver of HBV-ACLF patients was significantly enhanced, suggesting that NF-κB may also play an important role in the pathogenesis of HBV-ACLF.

Cylindromatosis (CYLD) is a known deubiquitinating enzyme (DUB), which removes ubiquitin chains from specific substrates, and has been shown to be a key negative regulator of NF-κB signalling pathway [14–16]. The removal of the K63-linked polyubiquitin chain from TNF receptor associated factor 2 (TRAF2) by CYLD leads to the inhibition of the IkB kinase (IKK) complex, stabilization of the NF-ĸB inhibitor IkBα, and retention of the classic NF-ĸB heterodimer p65/p50 in the cytoplasm [16]. Hence, down-regulation of CYLD may lead to hyperactivation of NF-ĸB. CYLD is involved in regulating a variety of physiological and pathological processes, ranging from immune responses and inflammation to cell cycle control and osteoclastogenesis [17–19], and its mutation or dysregulation is crucial for the pathogenesis of several important diseases, including cancer, lung injury, and atherosclerosis [20–23]. In liver disease, CYLD acts as an important regulator of hepatocyte homeostasis by preventing progressive fibrosis, inflammation, tumor necrosis factor (TNF) production, and expansion of hepatocyte apoptosis towards the central veins [14,15]. The broad impact of CYLD in these chronic inflammatory diseases and its protecting role in liver further suggests its potential role in the pathogenesis of HBV-ACLF.

The purpose of this study was to elucidate the molecular mechanism underlying pathogenesis of liver in HBV-ACLF patients by transcriptome analysis. Our data suggested that down-regulated CYLD may participate in exacerbating liver injury in patients with ACLF by the enhancement of NF-ĸB-dependent up-regulation of chemokines.

## Methods

### Study design

**Figure 1.**
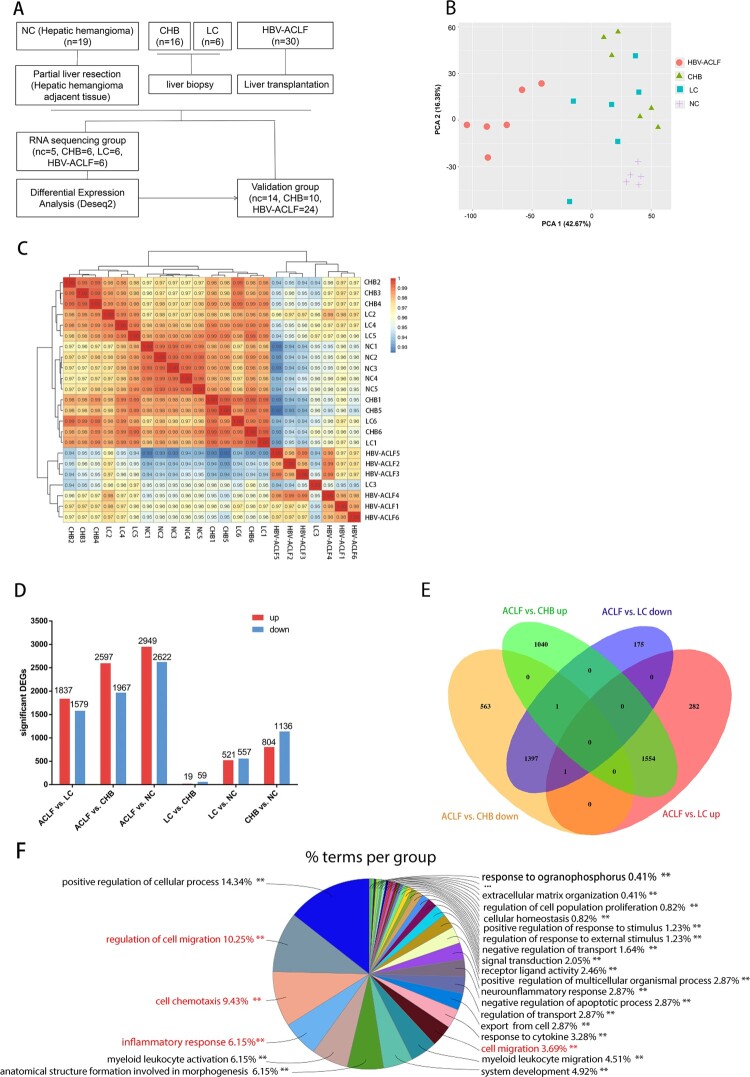
Transcriptomic characteristics of patients with HBV-ACLF. (A) Study design and patient group allocation. (B) Principal component analysis of subjects in the ACLF, LC, CHB and NC groups. (C) Hierarchical clustering analysis of subjects in the ACLF, LC, CHB and NC groups. (D) Number of DEGs analysed in pairwise comparisons among the four groups. (E) Venn diagram of the DEGs analysed in pairwise comparisons among subjects in the ACLF vs. CHB and ACLF vs. LC. (F) Biological pathways on ClueGO functional analyses from the overlapping up-regulated DEGs of ACLF vs. CHB and ACLF vs. LC. Abbreviations: ACLF, acute-on-chronic liver failure; CHB, chronic hepatitis B; DEGs, differentially expressed genes; HBV, hepatitis B virus; LC, liver cirrhosis; NC, normal controls.

A total of 30 HBV-ACLF patients who underwent liver transplantation at Huashan Hospital from June 2018 to Dec 2020 were enrolled in this study. Because HBV-ACLF patients in our study developed chronic hepatitis B and liver cirrhosis, we thus selected 20 chronic hepatitis B and 5 liver cirrhosis patients who received liver biopsy as controls, and 15 other hepatic hemangioma patients as normal controls. We performed RNA-seq analysis for the liver specimens of HBV-ACLF patients (*n* = 6), chronic hepatitis B (CHB) (*n* = 6), liver cirrhosis (LC) (*n* = 6), and normal control (*n* = 5). Liver specimens were obtained from patients with HBV-ACLF who received a liver transplant, while liver specimens from CHB and LC patients were obtained during biopsies. As a control, normal liver tissues were obtained from the normal liver tissue adjacent to the tumour from partial hepatectomy in patients with hepatic hemangioma. The patients and controls were randomly allocated into a sequencing group and a validation group (Figure 1(A)). Because the isolation of liver tissue samples from patients with liver cirrhosis was difficult, validation was not performed on samples from patients with liver cirrhosis. The use of blood and liver specimens was approved by the Ethics Committee of the Huashan Hospital, Fudan University (2018-251). Written informed consent was obtained from all participants. The main clinical and laboratory features of patients are presented in Supplementary Table 1, 2.

HBV-ACLF was diagnosed according to Chinese Group on the Study of Severe Hepatitis B (COSSH)-ACLF criteria, which is defined by acute deterioration of liver function and hepatic and/or extrahepatic organ failure in patients with HBV-related chronic liver disease, regardless of the presence of cirrhosis. COSSH-ACLF comprises three grades: ACLF-1, ACLF-2, and ACLF-3.

ACLF-1 includes patients: (1) with liver failure alone with an international normalized ratio (INR) ≥ 1.5 and/or kidney dysfunction and/or hepatic encephalopathy (HE) grade I or II; (2) with kidney failure alone; (3) with single type of organ failure of the coagulation, circulatory or respiratory systems and/or kidney dysfunction and/or HE grade I or II; and (4) with cerebral failure plus kidney dysfunction. ACLF-2 includes patients with failures of two organ systems and ACLF-3 includes patients with failures of 3 or more organ systems.

The definitions of organ failure, chronic hepatitis B, cirrhosis, are described in the Supplementary Methods S1.

### Functional synergy analysis for RNA sequencing

False discovery rate (FDR) and log2 Foldchange (log2 FC) were used as the key indicators for screening significantly differentially expressed genes (DEGs). To visualize the variance between samples, a principal component analysis (PCA) plot was generated using the plot PCA function in DESeq2 and visualized using ggplot2 (v3.1.0).

Gene ontology (GO) is an international standard system used to classify gene functions. It divides gene functions into three aspects: molecular function (MF), cell composition (CC), and biological process (BP). Kyoto Encyclopedia of Genes and Genomes (KEGG) is a set of artificially drawn pathway maps representing molecular interactions and reaction network [24]. We performed GO and KEGG enrichment analysis on the overlapping co-upregulated DEGs (FDR < 0.01 and log 2 FC > 1) between HBV-ACLF vs. CHB and HBV-ACLF vs. LC.

To identify the function of the DEGs, multiple bioinformatics analyses were performed using the Cytoscape ClueGO bioinformatics tool [25]. The overlapping co-upregulated and co-downregulated DEGs (FDR < 0.01 and log2 FC > 1.7) between HBV-ACLF vs. CHB and HBV-ACLF vs. LC were input into Cytoscope ClueGO software, the GO term fusion was used, and a threshold *p* ≤ 0.01 was set for enrichment. The GO biological process enrichment parameter “GO Tree Interval” was set to 3–6, the minimum gene of “GO Term Selection” was set to 3, the minimum gene proportion was set to 4%, and the kappa score was set to 0.4. After the selection parameters were run separately, the GO biological processes and their related target information were obtained.

We selected the chemotaxis-related genes between the co-upregulated DEGs (FDR < 0.01 and log2 FC > 1) of HBV-ACLF vs. CHB and HBV-ACLF vs. LC, imported into the STRING database, which is a database for predicting protein–protein interactions, to obtain the interaction relationship between the targets. The confidence level adopts the system default “score > 0.4,” saves it in the TSV format, and imports Cytoscape 3.5.1 software to build a more advanced protein interaction network diagram. A network analyser is used to calculate topology parameters, such as the node degree value, to filter the core targets.

### Cell lines and cell culture

THP-1 cells were obtained from Chinese Academy of Sciences Cell Bank (Shanghai, China) and cultured in RPMI1640 containing 10% FBS, penicillin (100 U/ml), and streptomycin (100 mg/ml) at 37°C with 5% CO_2_. Treatment THP-1 with Phorbol 12-myristate 13-acetate (PMA) (100 ng/ml) for 24 h to differentiate THP-1 into macrophage, then stimulated with lipopolysaccharide (LPS) (100 ng/ml), poly dA:dT(1ug/ml) and poly I:C(1 μg/ml), and collect cell lysates at different time point within 48 h.

### Transwell chemotaxis assay

The supernates (100 μl) from CYLD-depleted macrophages or control macrophages stimulated by LPS for 6 h were plated in the bottom chambers of Transwell plates (8 μm pore diameter, Sigma-Aldrich). Immune cells from normal human peripheral blood were obtained by lysing red blood cells, suspended in no FBS RPMI 1640 (2 × 10^5^ cells/100 μl), and then added to the upper wells of the Transwell system and incubated for 3 h at 37°C under 5% CO_2_. Immune cells migrated into the bottom chambers and the upper remaining cells were resuspended in PBS containing 5 mM EDTA and their absolute numbers were determined by flow cytometry. To determine the chemotactic index, we calculated a ratio of the number of immune cells that migrated into the bottom to the total number of immune cells [10].

Detailed information about mRNA sequencing, lentiviral transduction, western blot, RNA isolation, quantitative real-time polymerase chain reaction (RT-qPCR), flow cytometry, and histological experiment are described in Supplementary Methods S2.

### Statistical analysis

All statistical analyses were performed using Graphpad 6.0 (Graphpad Software, San Diego, CA) and SPSS software version 22 (SPSS, Chicago, IL, USA). Categorical variables were expressed as percentages (frequencies) and continuous variables were expressed as medians (interquartile ranges). Categorical variables were compared using the Chi-squared test or Fisher’s test. For continuous variables, the Kruskal–Wallis test was used to assess the characteristics of different groups of patients. Two-tailed *P* values were calculated, and the significance level was set at *P* < 0.05.

## Result

### Comparative transcriptome analysis of liver in HBV-ACLF patients

The clinical characteristics of 71 subjects participating in this study are shown in Supplementary Table 1 (RNA sequencing group) and Supplementary Table 2 (validation group). Liver specimens from 6 ACLF patients, 6 CHB patients, 6 LC patients, and 5 NC were subjected to transcriptomic sequencing. Potential molecular and mechanisms were explored by qRT-PCR, immunohistochemistry, and western blot in liver tissues from remaining 48 subjects (Figure 1(A)). The PCA show that patients with HBV-ACLF were clustered together and were separated from the other three groups patients (Figure 1(B)). The hierarchical clustering result show that ACLF patients were clustered together(Figure 1(C)). The pairwise differential expression analysis illustrated that there were significant differences between ACLF and other groups, while CHB had a small amount of DEGs compared with the LC group (Figure 1(D)). Considering that HBV-ACLF is developed from LC and CHB, we focused on comparing the overlapping DEGs between HBV-ACLF vs. LC and HBV-ACLF vs. CHB. Compared with LC, ACLF has 1836 up-regulated DEGs, of which 1554 DEGs overlapped with the up-regulated DEGs from the comparation of ACLF and CHB. Among the 1597 down-regulated DEGs, 1397 DEGs overlapped with the down-regulated DEGs from the comparation of ACLF and CHB (Figure 1(E,F)). These overlapped up-regulated DEGs and down-regulated DEGs were likely to be important genes involved in the pathogenesis of ACLF.

The overlapped co-upregulated DEGs between HBV-ACLF vs. CHB and HBV-ACLF vs. LC were inputted into Cytoscope ClueGO software. As expected, the identified terms were mostly involved in positive regulation of the cellular process, cell migration (e.g. regulation of cell motility, leukocyte migration and chemotaxis), cell chemotaxis (e.g. chemokine activity, response to chemokine, monocyte chemotaxis, and granulocyte chemotaxis), and inflammatory response (e.g. response to stress, cytokine secretion, defense response to bacterium) (Figure 1(E) and Supplementary Figure 1). The result of ClueGO analyses of these down regulated DEGs (HBV-ACLF vs. CHB overlap HBV-ACLF vs. LC) were involved in the organic acid metabolic process, organic hydroxy compound metabolic process, monooxygenase activity, and anion transport process (Supplementary Figure 2).

**Figure 2.**
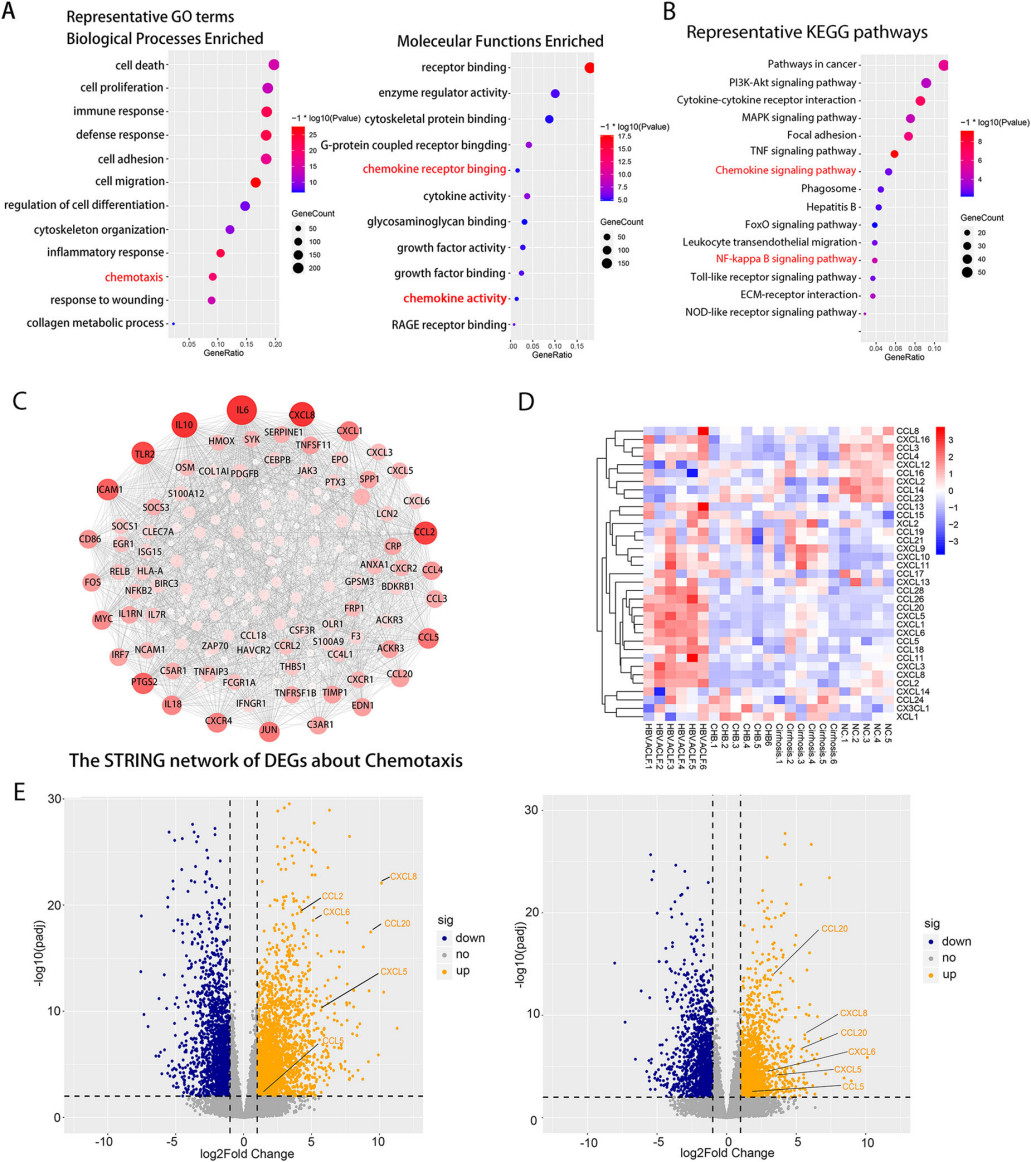
Transcriptome sequencing data analysis indicated the important role of inflammation and chemotaxis in the development of HBV-ACLF. The overlapping up-regulated DEGs of ACLF vs. CHB and ACLF vs. LC were analyzed for enrichment of (A) GO molecular functions and (B) KEGG pathways. (C) Protein–protein association network of DEGs related to chemotaxis from the overlapping up-regulated DEGs of ACLF vs. CHB and ACLF vs. LC. (D) Heatmap of chemokine and chemokine receptor genes among ACLF, LC, CHB and NC groups. (E) The volcano plot showing the expression of the key chemokines (CXCL8, CCL2, CCL5, CCL20, CXCL5, CXCL6) associated with ACLF between HBV-ACLF vs. CHB (left) and HBV-ACLF vs. LC (right). Abbreviations: ACLF, acute-on-chronic liver failure; CHB, chronic hepatitis B; DEGs, differentially expressed genes; HBV, hepatitis B virus; LC, liver cirrhosis; NC, normal controls; GO: gene ontology; KEGG: Kyoto Encyclopedia of Genes and Genomes.

Next, we performed gene annotation (GO) enrichment analysis on the overlapping up-regulated DEGs between HBV-ACLF vs. CHB and HBV-ACLF vs. LC. The results were consistent with that of ClueGO. GO enrichment analysis highlight the cell death and immune response among the top hits of broad transcriptional changes in the liver of HBV-ACLF patients (Figure 2(A)). Other significantly upregulated genes encoded molecules involved in cell proliferation, cell adhesion, cell migration, inflammatory response, and chemotaxis. Chemotaxis is of particular interest because cell migration, inflammation, and immune responses are all involved in chemotaxis, and the ClueGO analysis also suggests the enrichment of the chemotaxis pathway. KEGG analysis on the overlapping up-regulated DEGs between HBV-ACLF vs. CHB and HBV-ACLF vs. LC showed the up-regulated expression of the chemokine signaling pathway and NF-ĸB pathway, which are involved in chemokine production, providing supportive evidence for the involvement of NF-ĸB-dependent up-regulation of chemokines in hyperinflammatory response in liver tissue of HBV-ACLF patients (Figure 2(B)) [10–12].

We screened chemotaxis-related genes among the up-regulated DEGs of HBV-ACLF vs. CHB and HBV-ACLF vs. LC, and constructed a PPI network. As shown in Figure 2(C), the up-regulated PPI network included 75 nodes and 699 edges. According to degree levels, the top chemokine hub nodes were CXCL8, CCL2, CCL5, CCL20, CXCL1, CCL4, CCL3, CCL28, CCL18, CXCL3, CXCL5, and CXCL6. Figure 2(D) shows the heatmap of transcription levels of chemokines between groups. Based on the combined results of PPI and heatmap, we sought to focus on assessing the role of CXCL8, CCL2, CCL5, CCL20, CXCL5, and CXCL6 in HBV-ACLF patients (Figure 2(E), Supplementary Table 3). The function of these chemokines is listed in Supplementary Table 4. T lymphocytes, neutrophils, macrophage, monocytes, and NK cell are the major target cells [26].

### The comparison of transcriptomic data between ACLF-1 and ACLF-2/3.

**Figure 3.**
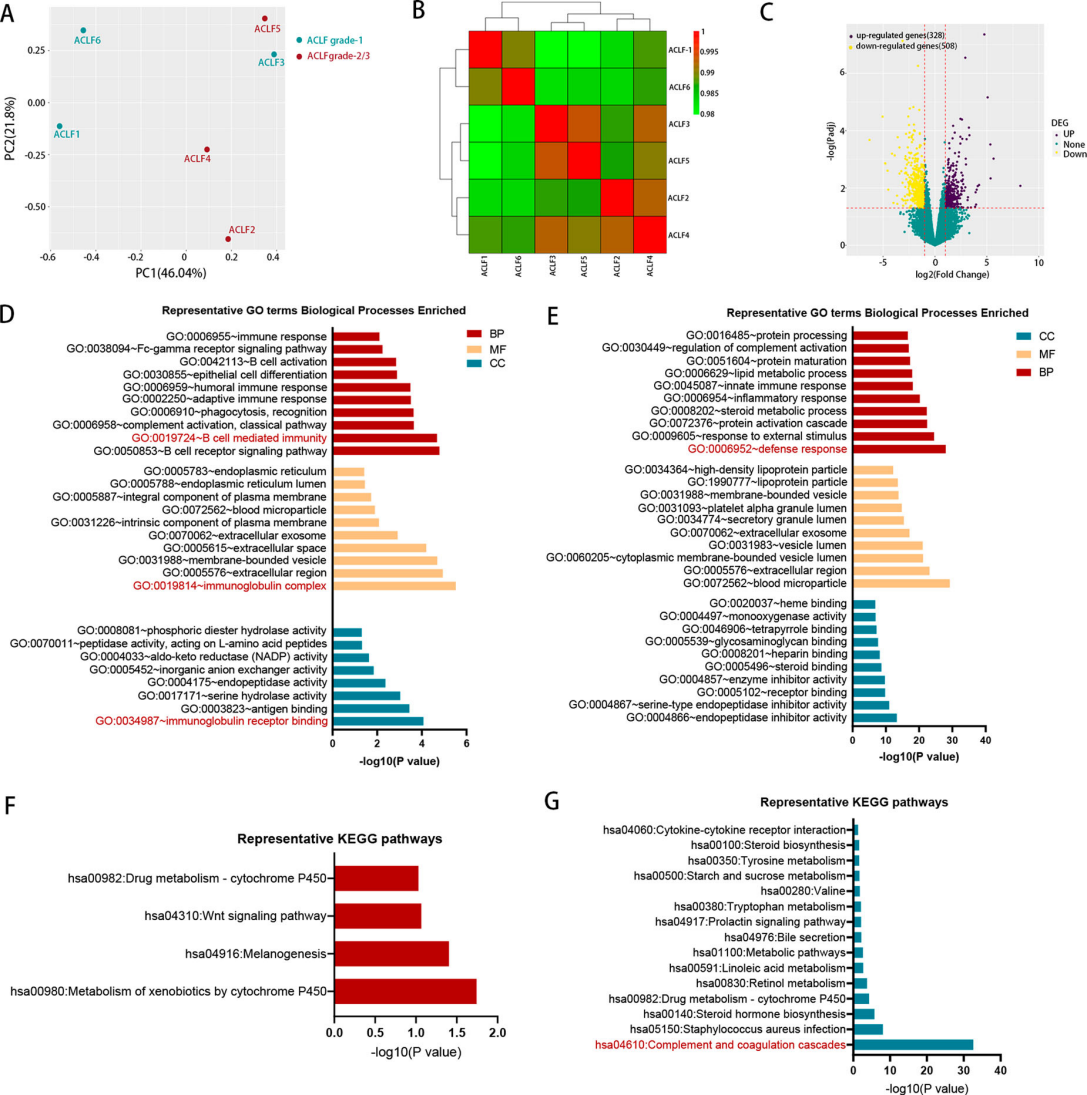
The comparison of transcriptomic data between ACLF-1 and ACLF-2/3. (A) Principal component analysis of subjects in the ACLF-1 and ACLF-2 groups. (B) Hierarchical clustering analysis of subjects in theACLF-1 and ACLF-2 groups. (C) The volcano plot between ACLF-1 and ACLF-2/3. GO enrichment analysis of the up-regulated DEG (D) and down-regulated DEG (E). KEGG enrichment analysis of the up-regulated DEG (F) and down-regulated DEG (G). Patients 1, 3, and 6 belong to ACLF-1, and patients 2, 4, and 5 belong to ACLF-2., Abbreviations: ACLF, acute-on-chronic liver failure; GO: gene ontology; KEGG: Kyoto Encyclopedia of Genes and Genomes; DEGs, differentially expressed genes;

HBV-ACLF patients can be classified into three grades (ACLF-1, ACLF-2, ACLF-3) according to their diagnosis criteria. Patients diagnosed with higher grade had more organ failure and worse prognosis [3]. To further study the mechanism of HBV-ACLF, we compared the transcriptomic characterization of ACLF-1 and ACLF-2. The PCA and hierarchical clustering result showed that ACLF-1 was separated from ACLF-2 (Figure 3(A,B)). Compared with ACLF-1, there were 328 up-regulated genes and 508 down-regulated genes in the ACLF-2 group (Figure 3(C)). GO enrichment analysis was performed in the up-regulated DEGs (Figure 3(D)) and down-regulated DEGs (Figure 3(E)), respectively. GO enrichment analysis of up-regulated DEG indicated B-cell mediated immunity played an important role in the development of ACLF-2 while the analysis of down-regulated DEG showed a weakened defense response in ACLF-2, which suggested that ACLF-2 patients might be more susceptible to infection. KEGG analysis of up-regulated DEGs (Figure 3(F)) and down-regulated DEGs (Figure 3(G)) showed a marked impairment of the complement and coagulation cascades in ACLF-2 patients, which was consistent with the fact that the majority of ACLF-2 patients were complicated with coagulation failure [3].

### Inflammation and chemotaxis associated with ACLF development

**Figure 4.**
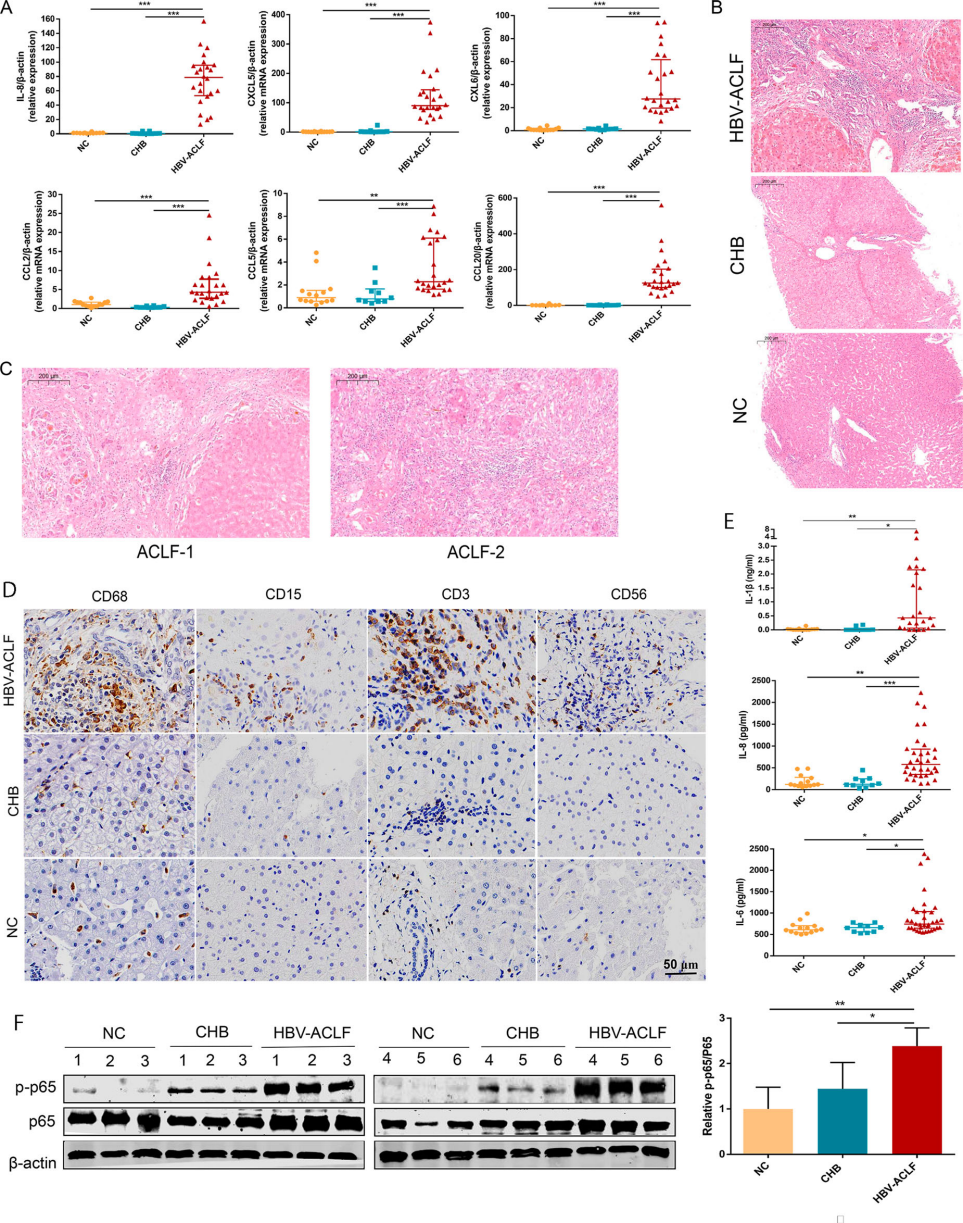
The liver of HBV-ACLF patients exhibited significantly increased chemokines, a large number of inflammatory cell infiltration and high activation of the NF-ĸB pathway. (A) The qRT-PCR validation of 10 key chemokine genes (*n* = 24/10/14, ACLF/CHB/NC groups, respectively). (B,C) Liver HE staining in patients from the HBV-ACLF, CHB, and NC groups. (D) Immunohistochemical staining of T lymphocytes (CD3), macrophages (CD68), neutrophils (CD15), and NK cells (CD56) in the liver of patients from the HBV-ACLF, CHB, and NC groups. (E) ELISA results of plasma IL-6, IL-8, and IL-1β in HBV-ACLF, CHB patients, and NC. Plot, medians with (p25, p75). The Kruskal–Wallis test, *p < 0.05, **p < 0.01 and ***p < 0.001. (F) Immunoblot analysis of HBV-ACLF, CHB, and NC livers for p-P65 and P65. Abbreviations: ACLF, acute-on-chronic liver failure; CHB, chronic hepatitis B; DEGs, differentially expressed genes; HBV, hepatitis B virus; LC, liver cirrhosis; NC, normal controls; HE; RT-PCR;

The pathogenesis of HBV-ACLF injury is complex and we decided to focus on inflammation and the chemotaxis process in our study, which accounted for the largest proportion of the DEGs among the up-regulated genes in HBV-ACLF. The six key chemokines (CXCL8, CCL2, CCL5, CCL20, CXCL5, and CXCL6) associated with ACLF progression were validated by qRT-PCR in the liver of CHB, ACLF patients, and NC, and the expression of all of these chemokine genes was significantly increased in the liver of ACLF patients, consistent with the gene expression detected in the sequencing data (Figure 4(A)). These chemokines attract a large number of inflammatory cells into the liver, exacerbating liver inflammation and damage. Hematoxylin–eosin(HE) staining showed that a large number of inflammatory cells infiltrated the liver of ACLF patients, and the infiltration of inflammatory cells in liver tissues of ACLF-2/3 patients were more prominent than that of ACLF-1 patients (Figure 4(B,C)). Neutrophils, T lymphocytes, NK cells, and monocytes are the target cells of these chemokines, and the number of these cells significantly increased in the liver of HBV-ACLF patients (Figure 4(D)). Similar with previous data, systemic inflammation was the primary cause of organ failures in ACLF, serum pro-inflammatory cytokines (IL-6, IL-8, IL-1β) were significantly elevated in HBV-ACLF patients (Figure 4(E)). Interestingly, majority of these cytokines and chemokines (IL-1β, IL-6, IL-8, CCL2, CCL5, CCL20, CCL20, CXCL5, and CXCL6) are known as NF-ĸB targeted genes [10–12]. In addition, KEGG analysis showed the activation of the NF-ĸB pathway in the liver tissues of HBV-ACLF patients (Figure 2(B)), and after assessing NF-ĸB pathway activation in livers from CHB, ACLF patients and NC, a robust increase in phospho-p65 was observed in the livers from 6 HBV-ACLF patients, but not in CHB patients or NC, supporting the involvement of the activated NF-ĸB pathway in HBV-ACLF (Figure 4).

### Down-regulation of CYLD in macrophage caused hyperactive NF-κB signalling, higher levels of chemokines and cytokines secretion, and increased inflammatory cells infiltration

**Figure 5.**
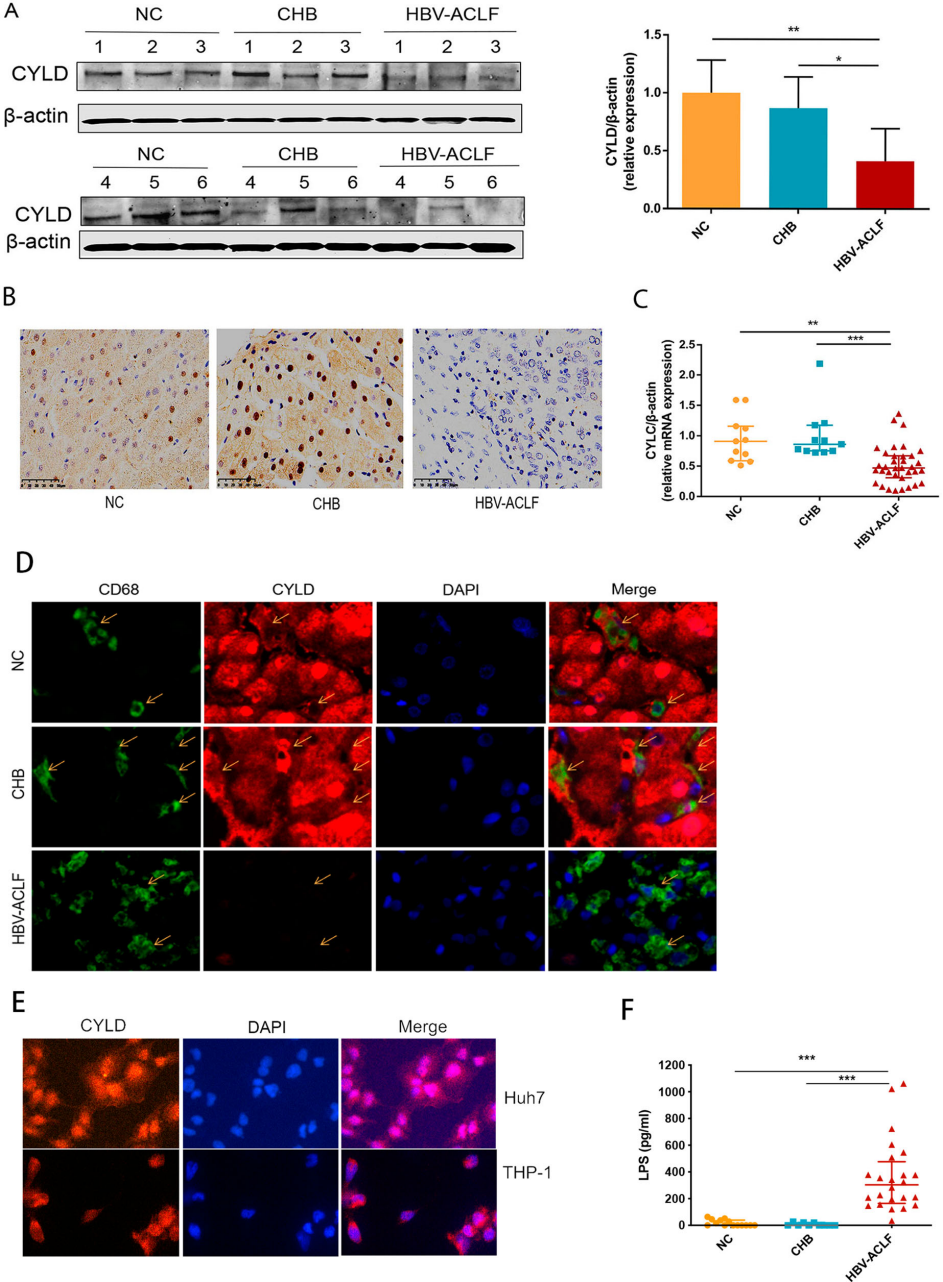
CYLD expression in liver and PBMC was down-regulated in HBV-ACLF patients. (A) Immunoblot analysis of HBV-ACLF, CHB, and NC livers for CYLD. (B) Immunohistochemical staining of CYLD in the liver of patients from the HBV-ACLF, CHB, and NC groups. (C) The qRT-PCR of CYLD in the PBMC of patients from the HBV-ACLF, CHB, and NC groups. (D) Immunohistochemical staining of CYLD and CD68 in the liver of patients from the HBV-ACLF, CHB, and NC groups. (E) Immunohistochemical staining of CYLD in Huh7 and THP-1. (F) ELISA results of plasma LPS in HBV-ACLF, CHB patients, and NC. Plot, medians with (p25, p75). The Kruskal–Wallis test, *p < 0.05, **p < 0.01 and ***p < 0.001. Abbreviations: Cylindromatosis (CYLD), ACLF, acute-on-chronic liver failure; CHB, chronic hepatitis B; DEGs, differentially expressed genes; HBV, hepatitis B virus; LC, liver cirrhosis; NC, normal controls; PBMC, peripheral blood mononuclear cell; LPS, lipopolysaccharide.

We next investigated the mechanism underlying NF-ĸB activation. Macrophages are crucial in the pathogenesis of ACLF among the variety types of cells that compose liver, which drives the initiation, progression, and resolution of injury and related inflammation [27]. Activated macrophages secrete pro-inflammatory cytokines, reactive oxygen species, and chemokines that amplify pro-inflammatory signal and increase the recruitment of inflammatory cells into the liver, thereby enhancing the inflammatory process [28]. The highest proportion of infiltrating innate immune cells in the liver are macrophages in HBV-ACLF patients (Figure 4(D)), among which a large proportion of macrophages in the liver of ACLF patients are derived from peripheral monocytes [27] and capable of secreting chemokines and cytokines. Therefore, we carried out RNA-seq analysis for the monocytes from HBV-ACLF and chronic hepatitis B patients (Supplementary Figure 3). KEGG enrichment analysis of monocyte RNA-seq also revealed NF-kB pathway activation in monocytes of HBV-ACLF patients. Thus, we further compared the expression of genes in the core NF-kB pathway [29], and found that CYLD, CARD11, NF-KBIA, and NFKB2 were down-regulated in the monocytes of HBV-ACLF (Supplementary Figure 3). CYLD is primarily a negative regulator of NF-κB signalling and plays a key role in inflammation [16]. In addition, CYLD is a protective molecule against liver disease which contributes to hepatic homeostasis and restoration upon liver injury [15–18]. CYLD-NF-κB might play an important role in hyperinflammatory response in HBV-ACLF patients. We thus evaluated the expression level of CYLD in the liver and peripheral blood mononuclear cells (PBMCs) of patients with ACLF, CHB, and NC. Liver immunohistochemistry and western blot analysis showed that the expression of CYLD was significantly down-regulated in HBV-ACLF patients compared with CHB and NC patients (Figure 5(A,B)). Moreover, the mRNA expression level of CYLD in PBMC of HBV-ACLF patients was significantly lower than that of CHB patients and NC patients (Figure 5(C)). Liver immunohistochemistry showed CYLD was widely expressed in many types of cells in CHB patients and normal control. Among which, CYLD was mainly expressed in the nucleus of hepatocytes, with some expression in the cytoplasm. For macrophages, CYLD also seemed to be expressed in the cytoplasm. Downregulation of CYLD expression in the liver of HBV-ACLF patients involves all liver cells. To further confirm the expression of CYLD in liver macrophages, we performed immunofluorescence double staining, and found the expression level of CYLD in liver macrophages of HBV-ACLF patients was significantly lower than that of CHB patients and NC patients (Figure 5(D)). The relative higher expression of CYLD in hepatocyte nuclei of control group is an interesting phenomenon. The mechanism about CYLD had always been focused on cytoplasm previously [14–23,30]. Even though some articles showed CYLD expressed in nuclear, the mechanism remained unclear [31,32]. For this reason, we did the immunofluorescence of CYLD for THP-1 (monocyte cell line) and Huh7 (hepatocyte cell line) respectively, and found that the result was consistent with tissue sections, in which CYLD was mainly located in the nucleus in hepatocyte and cytoplasm in monocytes (Figure 5(E)). In this study, we hoped to describe the regulation of macrophage chemotaxis and inflammation, so we utilized monocyte/macrophage cell line (THP-1) to investigate the mechanism underlying CYLD regulation of inflammation and chemotaxis.

### CYLD enhanced the inflammatory response and chemokines production in LPS-stimulated THP-1 cells

**Figure 6.**
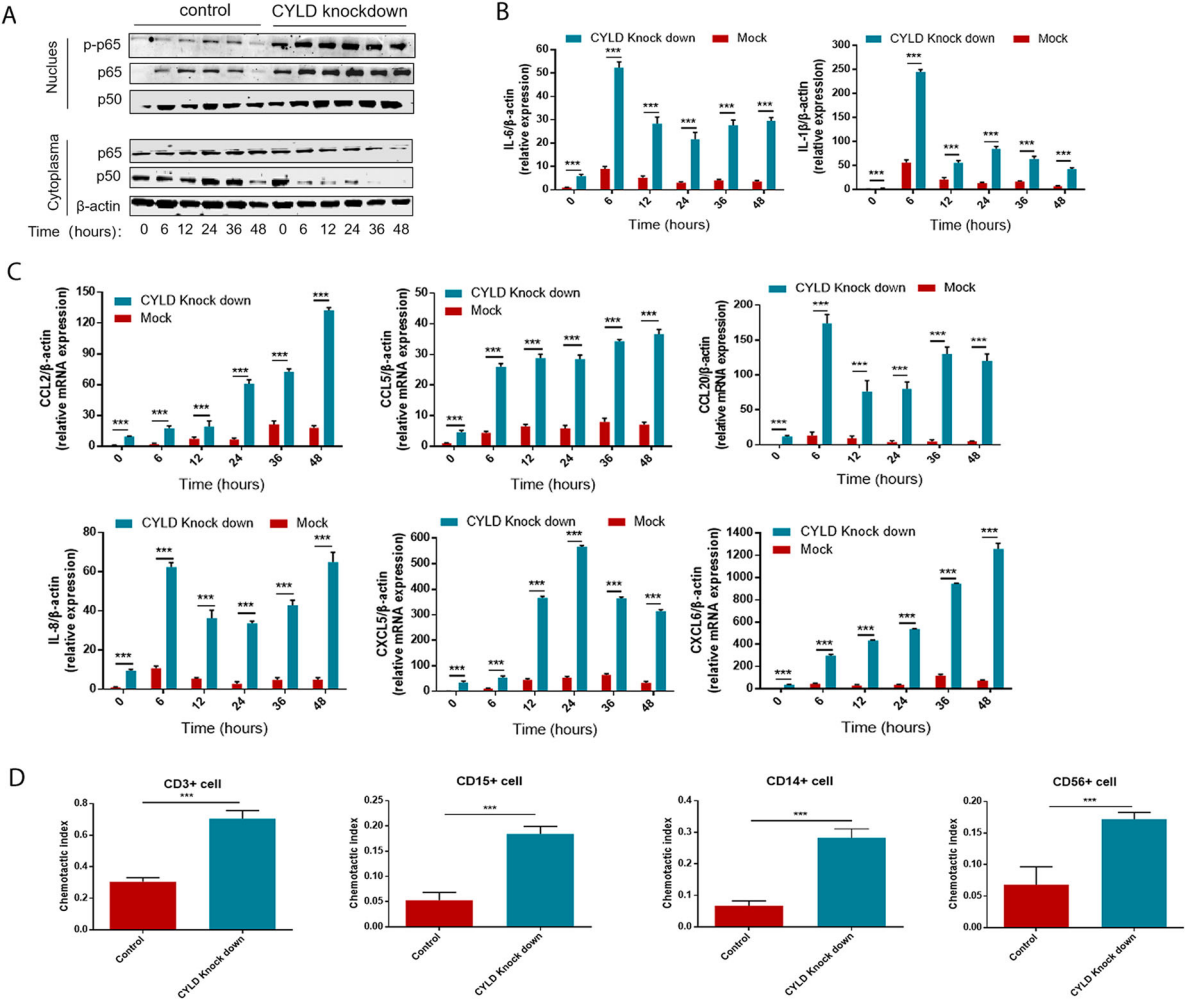
Depletion of CYLD enhanced inflammatory response by enhancing NF-ĸB activation. THP-1 (8 × 10^5^ cells/ml) were seeded in 6-well cell culture plate added with PMA (100 ng/mL) for 24 h, then were stimulated with LPS (100 ng/mL) and lysed for collecting cell lysates and supernates at different time point within 48 h. Protein levels of p65, p50 and Phospho-p65 were detected in the nucleus and cytoplasm, respectively. CYLD knockdown in THP-1 significantly enhances NF-ĸB activation (A), increases the production of pro-inflammatory cytokines (B) and chemokine (C). Supernates from CYLD-depleted macrophages stimulated by LPS for 6 h exhibited an enhanced chemotactic migratory effect on immune cells from normal human peripheral blood (D). Values are shown as fold change from control and expressed as the mean, *n* = 3 (a single sample analysed in triplicate); bars show SD. *P < 0.05, **P < 0.01, ***P < 0.001. Abbreviations: PMA, Phorbol 12-myristate 13-acetate; LPS, lipopolysaccharide. CYLD, Cylindromatosis.

To investigate the regulatory role of CYLD in inflammation and chemotaxis, we generated CYLD knockdown THP-1 cells by lentivirus transduction. HBV flares are an important cause of HBV-ACLF [3], and their HBV DNA replication levels are also very high (Tables s1, s2). Besides, most ACLF patients have gut microbiota translocation [33,34], and LPS was significantly elevated in HBV-ACLF patients (Figure 5(F)). Therefore, we chose poly dA:dT (viral DNA analog), poly I:C (viral RNA analog), and LPS as stimuli to study the expression of cytokines and chemokines in macrophages. In LPS-stimulated experiments, translocation of P50/65 from the cytoplasm to the nucleus and phosphorylation of NF-ĸB/P65 in the nucleus was significantly increased in CYLD knockdown THP-1 cells and sustained for 48 h (Figure 6(A)). Consistent with activation of the NF-ĸB pathway, the chemokines (CCL2, CCL5, CCL20, CXCL5, CXCL6 and CXCL8) were significantly increased in the liver of HBV-ACLF patients were marked increased in CYLD-depleted THP-1 cells, and remained elevated at 48 h. Of note, some pro-inflammatory cytokines (IL6, IL-1β) were also increased in CYLD-depleted THP-1 cells, which was also consistent with previous studies (Figure 6(B,C)). On the other hand, we used Poly dA:dT and Poly I:C as stimuli and confirmed that knockdown of CYLD could enhance activation of the NF-KB pathway. The basal expression levels of cytokines (IL-6, IL-1β) and chemokines (CCL2, CCL5, CCL20, CXCL5, CXCL6, CXCL8) were higher in CYLD-knockdown THP-1 cells, but Poly dA:dT or Poly I:C stimulation failed to significantly further enhance their expression in both control and CYLD-knockdown THP-1 cells (Supplementary Figure 4). Therefore, the elevation of cytokines and chemokines in HBV-ACLF patients may be mainly related to molecule of bacterial origin such as LPS. So we chose to treat THP-1with LPS in further experiments.

**Figure 7.**
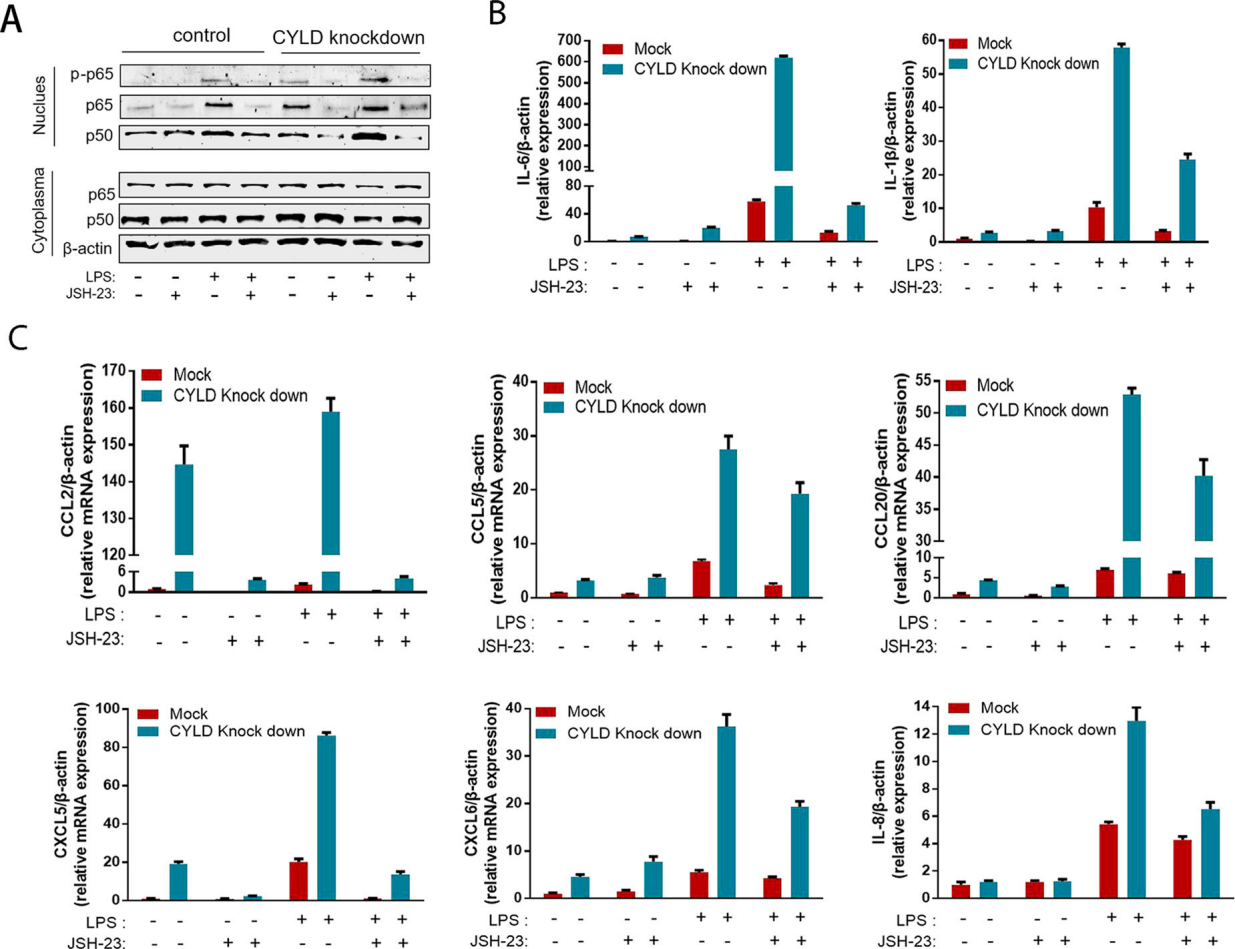
Blocking NF-ĸB in THP-1 attenuated the excessive inflammatory response caused by CYLD downregulation. THP-1 (8 × 10^5^ cells/ml) were seeded in 6-well cell culture plate added with PMA (100 ng/mL) for 24 h, then JSH-23 (an inhibitor of NF-κB nuclear translocation, 20 μM) was then added 3 h before stimulation with LPS (100 ng/mL). Cell lysates and supernates were collected at 6 h after LPS stimulation. Phospho-p65, p50 and p65 of nuclear and cytoplasmic fractions were detected respectively. NF-ĸB activation was suppressed in both CYLD-depleted and control THP-1 cells (A). The majority of chemokines (CCL2, CCL5, CCL20, CXCL5, CXCL6 and CXCL8) and cytokines (IL-6 and IL-1β) were suppressed in both CYLD-depleted and control THP-1cells. Values are shown as fold change from control and expressed as the mean, *n* = 3 (a single sample analysed in triplicate); bars show SD. Abbreviations: CYLD, Cylindromatosis; PMA, Phorbol 12-myristate 13-acetate; LPS, lipopolysaccharide.

Neutrophils, T lymphocytes, NK cell, macrophage, and monocytes are the major target cells of CCL2, CCL5, CCL20, CXCL5, CXCL6, and CXCL8. Therefore, we investigated the role of CYLD in regulating chemotactic migration of inflammatory cells (neutrophil marker CD15, monocyte marker CD14, NK cell marker CD56, and T lymphocyte marker CD3). Deficiency of CYLD enhanced chemotactic migration of neutrophils, monocytes, and lymphocytes by macrophages (Figure 6(D), Supplementary Figure 5). Inhibition of NF-ĸB by 2-diamine (JSH-23, an inhibitor of NF-κB nuclear translocation), attenuated the increased chemokines and cytokines expression (Figure 7) [35].

## Discussion

To our knowledge, this study is the first to provide a comprehensive view of the landscape of the transcriptional profile in the liver of patients with HBV-ACLF. The analysis and profiling of up-regulated genes in the HBV-ACLF patients indicated that cell death, immune response, inflammatory response, and chemotaxis are involved in the development of HBV-ACLF. While down-regulated genes were mostly involved in metabolic processes, especially for the organic acid metabolic process and organic hydroxy compound metabolic process. Previous studies have found that the changes of metabolic processes in peripheral blood were associated with the development of ACLF [36–38]. The blood metabolomic database of the consortium acute on chronic liver failure study (CANONIC study) indicated that intense systemic inflammation of ACLF patients was associated with blood metabolite accumulation and profound alterations in major metabolic pathways [36]. A recent research performed transcriptomics analysis of PBMC from HBV-ACLF patients and observed prominent metabolic alterations (including lipid metabolism, fatty acid metabolism, autophagy and oxygen homoeostasis) across all stages of ACLF development [37]. Here, we demonstrated that changes of metabolism process in liver are also important in the development of ACLF. In addition, patients with higher grade HBV-ACLF show higher activation of B-cell mediated immunity, weakened defense response, and marked impairment of the complement and coagulation cascades. The above transcriptomic features were consistent with the clinical features of the patients. The coagulation function of these patients with ACLF-2/3 grades was worsened, and the proportion of bacterial infections in ACLF-2/3 patients was increased. Another interesting point is that many pathways enriched by GO analysis among up-regulated genes are related to B cell-mediated immunity. Few studies have reported the role of B cells in ACLF, but it is likely to be related to the progression of ACLF according to our study, which can provide a good idea for the follow-up study of the mechanism of ACLF.

Sequencing data analysis showed that among the genes up-regulated in ACLF, genes related to inflammatory response and chemotaxis accounted for the largest proportion of the up-regulated DEGs. Systemic inflammation is known as the primary cause of organ failures in ACLF [39–41]. Liver inflammation is mediated by chemokines, which regulate the migration and activities of hepatocytes, Kupffer cells, hepatic stellate cells, endothelial cells, and circulating immune cells [26]. We thus investigated the mechanism underlying the chemotaxis and inflammatory response and identified CCL2, CCL5, CCL20, CXCL5, CXCL6, and CXCL8 as key chemokines in the pathogenesis of HBV-ACLF patients. These chemokines attracted neutrophils, T lymphocytes, monocytes, and NK cell migrating to liver, which in turn exacerbated liver inflammation and damage. Interestingly, the number of inflammatory cells in the liver of HBV-ACLF was also significantly higher than that in CHB patients or NC patients, and severe patients (ACLF-2 or ACLF-3) exhibited more pronounced inflammatory cell infiltrates than mild ones (ACLF-1). Furthermore, our data revealed activation of NF-ĸB, a key regulator of many chemokines and inflammatory factors in the liver of patients with HBV-ACLF, leading to excessive inflammation in HBV-ACLF patients.

These findings prompted us to further investigate the molecular mechanism underlying the activation of NF-ĸB. CYLD is known as an important negative regulator of the NF-ĸB pathway via deubiquitination of TAK1, TRAF2, and TRAF6. Down-regulation of CYLD leads to the overactivation of the NF-ĸB pathway, which in turn increases the up-regulation of NF-ĸB dependent target genes including chemokines [42,43]. Moreover, CYLD is an important liver protective gene, which plays an important role in the pathogenesis of chronic liver disease [14,15]. However, the role of CYLD and the underlying mechanisms in HBV-ACLF patients still remain largely unknown. In our study, CYLD was down-regulated in peripheral monocytes, PBMC, and the liver of HBV-ACLF patients. Previous studies have shown that CYLD is ubiquitously expressed in hepatocytes, cholangiocytes, Kupffer cells, and hepatic stellate cells (HSCs) in liver tissue [15,44]. Down-regulation of CYLD expression was observed in all liver cells in liver from HBV-ACLF patients. Hepatocyte-specific disruption of CYLD leads to spontaneous and progressive hepatocyte apoptosis, Kupffer cell activation, inflammatory cell infiltration, TNF production, and NF-kB Activation [14]. CYLD controls hepatocyte growth factor (HGF) expression in activated HSCs, which in turn affects liver fibrosis and inflammation [45]. As for macrophage, the inhibition of NF-κB by CYLD could reduce IL-6 production and ROS synthesis [46]. The function of CYLD in various cell types of the liver could also regulate inflammation. It is worth noting that in the liver of the control group, CYLD was located mainly in the nucleus for hepatocytes and the cytoplasm for macrophages. The change of CYLD in hepatocytes nucleus is an intriguing phenomenon. Previous studies have proved that the expression level of CYLD in the nucleus was related to the prognosis of hepatocellular carcinoma, but the mechanism was unclear [31,32]. Almost all studies on the pathogenesis of CYLD are focused on the cytoplasm [14–23] but the role of CYLD in the nucleus is to be understood. The mechanism about down-regulation of CYLD in the hepatocyte nucleus of HBV-ACLF patients will be our future research direction.

Previous studies indicated that CYLD regulates chemokine production [42,47], and down-regulated CYLD leads to up-regulation of CCL2 and IL-8 production and exacerbates inflammatory response [42]. In addition, CYLD regulates CXCL5 production by inhibiting NF-ĸB, thus affecting the migration of tumour-associated neutrophils [47]. However, the effect of CYLD on other chemokines has not been studied. This study revealed that CYLD regulates a number of key chemokines and pro-inflammatory cytokines (CCL2, CCL5, CCL20, CXCL5, CXCL6, CXCL8, IL-6, and IL-1β) that were increased in the liver of HBV-ACLF patients.

Knockdown of CYLD resulted in the sustained activation of NF-ĸB in macrophages, as well as sustained increase of chemokines and inflammatory cytokines. Blocking NF-ĸB pathway by JSH-23 attenuated the up-regulation of chemokines and pro-inflammatory cytokines induced by CYLD depletion, thereby suggesting that CYLD negatively regulates expression of CCL2, CCL5, CCL20, CXCL5, CXCL6, CXCL8, IL-6 and IL-1β via inhibition of NF-ĸB. We also demonstrated that CYLD regulates chemotactic migration of neutrophil, monocyte, T lymphocytes in macrophages, which was consistent with liver inflammatory cell infiltration in patients with HBV-ACLF.

Relapse or flare of HBV infection is one of the most important precipitating events in HBV-ACLF patients [3], however our data showed that the viral replication levels of HBV-ACLF patients were not so high. This may be due to HBV DNA levels in ACLF patients were recorded on the day of transplantation. In the presence of intense inflammation, massive hepatocyte necrosis and the application of antiviral drugs, HBV DNA levels decline very rapidly after the onset in HBV-ACLF patients [48]. Therefore, HBV-ACLF patients who underwent surgery after waiting for a period of time had decreased HBV DNA levels, although they indeed had very high HBV DNA levels at the time of onset.

Among the three stimuli (LPS, poly dA:dT, poly I:C) we selected for macrophage cell experiments, only LPS increased the production of cytokines and chemokines. Although most ACLF patients exhibited high replication level of HBV DNA, poly dA:dT and poly I:C had no significant effect on cytokine and chemokine expression. However most ACLF patients have gut microbiota translocation [33,34], LPS was significantly elevated in HBV-ACLF patients (Figure 5(F)). Besides, ClueGO analysis of up-regulated DEG in ACLF exhibited the process of response to molecule of bacterial origin (Supplementary Figure 1). Therefore, the elevation of cytokines and chemokines in HBV-ACLF patients may be mainly related bacterial components such as LPS.

**Figure 8.**
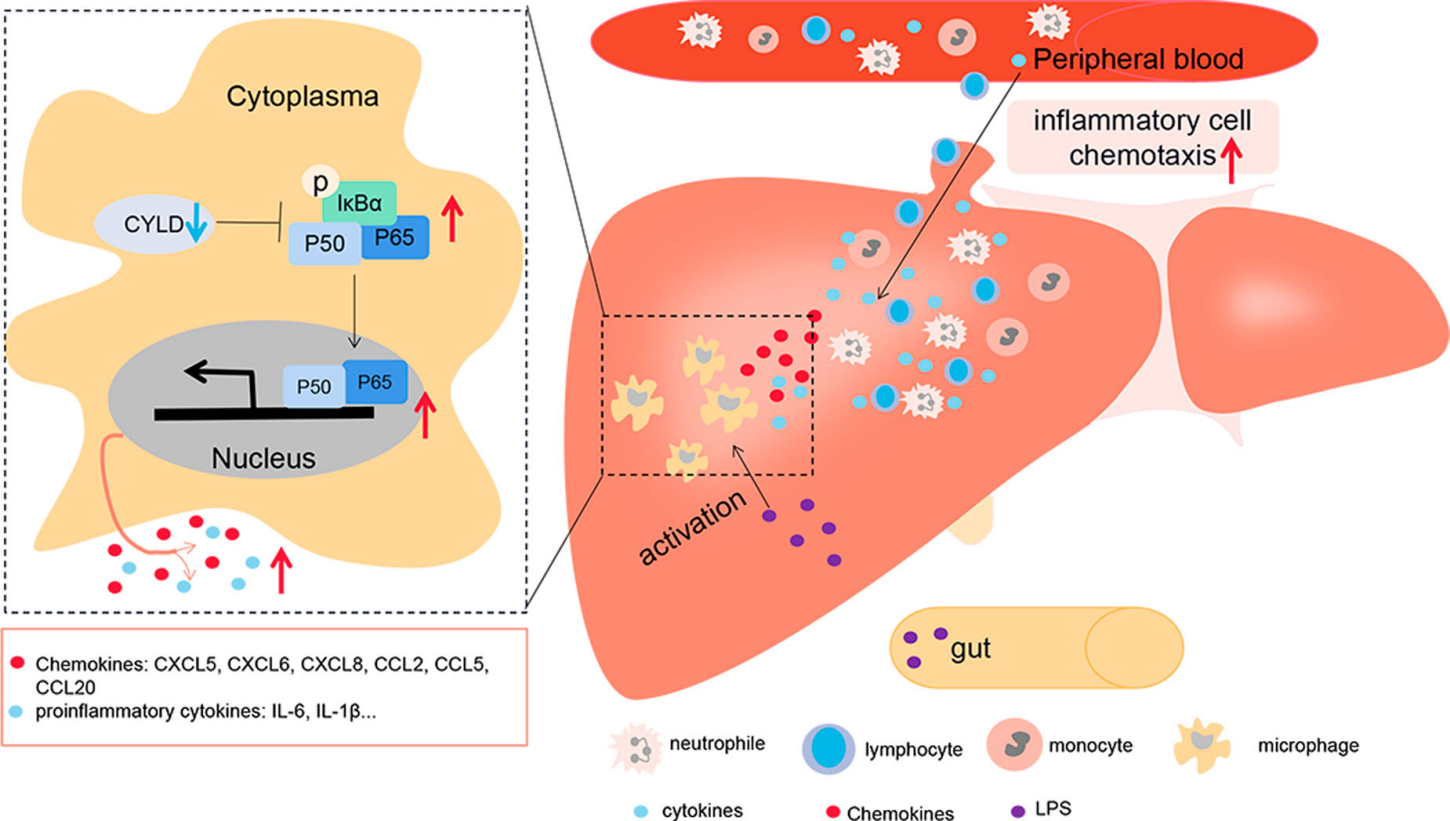
Illustration of how down-regulated CYLD induces a hyperinflammatory response in HBV-ACLF patient. PAMP from intestinal bacterial products (such as LPS) and DAMP molecules from necrotic hepatocytes activate intrahepatic macrophages, leading to the activation of NF-ĸB in macrophages. NF-ĸB activation remains sustained due to the down-regulated CYLD in HBV-ACLF patients, leading to high level of secretion of chemokines and pro-inflammatory factors. Chemokines attract a large number of inflammatory cells including lymphocytes, neutrophils, monocytes and NK cells to infiltrate the liver, thus aggravating tissue damage of the liver. Abbreviations: PAMP, pathogen-associated molecular patterns; DAMP, damage-associated molecular pattern; CYLD, Cylindromatosis; LPS, lipopolysaccharide; ACLF, acute-on-chronic liver failure; CHB, chronic hepatitis B; HBV, hepatitis B virus.

The current study provided supportive evidence to test our hypothesis for the possible mechanism underlying the injury in patients with HBV-ACLF: pathogen-associated molecular patterns (PAMPs) from intestinal bacterial products and damage-associated molecular patterns (DAMPs) molecules from necrotic hepatocytes activate intrahepatic macrophages [49], leading to the activation of NF-ĸB in macrophages. However, down-regulated CYLD in HBV-ACLF patients is unable to fully exert its anti-inflammatory role, leading to enhanced and sustained activation of NF-ĸB, and subsequent overproduction of a large number of pro-inflammatory mediators to aggravate tissue damage. The increased secretion of chemokines attracted a large number of inflammatory cells, including T lymphocytes, neutrophils, monocytes, and NK cells, to infiltrate the liver, further aggravating liver inflammatory damage (Figure 8).

In summary, we found that chemotaxis of various inflammatory cells in the liver was significantly enhanced in HBV-ACLF patients. CYLD negatively regulated the expression of chemokines and pro-inflammatory cytokines in macrophage, and down-regulated CYLD aggravates inflammatory cell chemotaxis through enhancing NF-κB activation in HBV-ACLF. However, the precise mechanism of how various types of inflammatory cells in the liver contribute to liver damage warrants further investigation. The role of CYLD in HBV-ACLF also needs to be further confirmed using animal models. Our results suggest that restoring the expression of CYLD may represent a novel therapeutic strategy for HBV-ACLF.

## Supplementary Material

Supplemental Material
