# Supplementary material for: Down-regulated cylindromatosis enhances NF-κB activation and aggravates inflammation in HBV-ACLF patients
Source: Emerg Microbes Infect. 2022 Jun 7;11(1):1586–601. doi: 10.1080/22221751.2022.2077128 (PMC9186363; doi:10.1080/22221751.2022.2077128)
Supplement: Supplemental Material [file TEMI_A_2077128_SM1449.doc]

**Supplementary materials:**

**Contents of Supplementary data**

| **contents** | **number** |
| --- | --- |
| **Supplementary methods** | **2** |
| **Supplementary Figures** | **5** |
| **Supplementary Tables** | **4** |

***Supplementary methods***

***Methods S1: Definitions***

*Organ failure*

liver failure was defined by a TBil level of ≥12.0 mg/dL; kidney failure was defined by a serum creatinine (Cr) level of ≥2.0 mg/dL or the use of renal replacement therapy; cerebral failure was defined as the presence of grade III or IV HE; coagulation failure was defined by an INR >2.5; circulatory failure was defined by the use of vasoconstrictors; respiratory failure was defined by a ratio of PaO2/FiO2 ≤ 200 or SpO2 to FiO2 ≤ 214.4.[1]

*Chronic hepatitis B*

The underlying liver disease was chronic hepatitis B, defined as the presence of hepatitis B surface antigen for more than 6 months, with or without cirrhosis. [2]

*Cirrhosis*

Cirrhosis was diagnosis based on previous liver biopsy results, clinical evidence of previous decompensation and laboratory tests, endoscopy (esophageal and gastric varices) and radiological imaging of portal hypertension and/or liver nodularity.^3,6^ Pathology: (a) Presence of parenchymal nodules; (b) Differences in liver cell size and appearance; (c) Fragmentation of the biopsy specimen; (d) Fibrous septa; (e) Altered architecture and vascular relationships. Depending on the size of the nodules, there are three macroscopic types: micronodular, macronodular, and mixed cirrhosis. Endoscopy：Esophageal and gastric varices. Radiology：Ultrasound: The liver may appear small and nodular, with increased echogenicity and irregular-looking areas; an enlarged caudate lobe, widening of the liver fissures and enlargement of the spleen. Other radiologic tests include elastography techniques, abdominal CT and liver/bile duct MRI (MRCP).[3]

**Methods S2:**

*Methods for mRNA sequencing*

Samples identified with high purity (1.8–2.0 range A260/280) and quality (RIN > 8.0) were then processed for library preparation. Using magnetic beads with Oligo (dT) to perform A-T base pairing and binding with polyA, mRNA can be isolated from total RNA for downstream library construction. Add fragmentation buffer to randomly fragment mRNA into small fragments of about 200bp. RNA fragments were then subsequently reverse transcribed into cDNA strands, followed by adapter ligation and library amplification. Sequencing of these libraries was performed on the Illumina Hiseq/Miseq platform.

*Lentiviral transduction*

CRISPR lentivirus was used to knockout CYLD in THP-1 cells. The oligoes targeting CYLD are as follows:

CYLD-Forward: CACCGATATTCAAGATCGTTCTGTG；

CYLD-Reverse: AAACCACAGAACGATCTTGAATATC

Lentiviruses were produced by co-transfecting 293T cells in a 6-well plate with, per well, 1 μg of pVSV-G (envelop-encoding plasmid), 2 μg of psPAX2 (packing plasmid), and 4 g of lentiCRISPRs directed against human CYLD or lentiCRISPR vector, in a 1-ml volume using lipo 2000 transfection reagent (Thermo Fisher Scientific, Waltham, MA). The lentivirus was harvested 48 h later, filtered through a 0.45 μm filter, and stored at 80°C until transduction. THP-1 cells were transduced with lentiviruses to generate CYLD knockdown THP-1 cells. The efficiency of transfection was confirmed by Western blotting

*Western blot*

# The nuclear and cytoplasmic fractions were separated according to the protocol of commercial kit (Thermo Scientific, USA). Protein concentration was determined by BCA protein assay kit (Thermo Fisher Scientific, USA), then the supernatant of 40 μg protein was subject to SDS-PAGE electrophoresis and transferred to a nitrocellulose blotting membrane (GE Healthcare Life Sciences, Gemany). 5% non-fat milk was used to block membranes for 1 h followed by the incubation of corresponding primary and appropriate secondary antibodies. The protein band was visualized by Odyssey infrared imaging system (LI-COR, USA).

# The Abs used in the current study were the following: CYLD Rabbit mAb, Phospho-NF-kB p65 (Ser536) Rabbit mAb, NF-kB p65 Rabbit mAb, and [β-actin Mouse mAb](https://www.cellsignal.cn/products/primary-antibodies/b-actin-8h10d10-mouse-mab/3700?site-search-type=Products&N=4294956287&Ntt=β-actin&fromPage=plp) were purchased from Cell Signaling Technology，Anti-NFkB p105 / p50 p50 [Rabbit mAb](https://www.cellsignal.cn/products/primary-antibodies/b-actin-8h10d10-mouse-mab/3700?site-search-type=Products&N=4294956287&Ntt=β-actin&fromPage=plp) were purchased from Abcam.

*RNA isolation and quantitative real-time polymerase chain reaction (RT-qPCR)*

Total RNA was isolated from each sample using trizol reagent (Sigma, USA). Complementary DNA (cDNA) was synthesized from total RNA with reverse transcription kit (Tiangen, China) according to the manufacturer’s instructions. RT-qPCR was performed using SYBR Green RealTime PCR Detection System (Tiangen, FP207, China). The total reaction volume was 20 µL and was prepared as follows: 10 µL of 2× FastFire qPCR Premix (Tiangen,China), 0.6 µL of each primer (10 µmol/L), 1 µL of cDNA template (0.5 ng/µL), and 7.8 µL of ddH2O. The cycling conditions were as follows: Predegeneration was performed at 95 °C for 1 min , followed amplification was performed 40 cycles at 95°C for 5sec, 60°C for 10 sec, and 72 °C for 15 sec. Transcript levels were normalized vs. β-actin expression. The gene expression was calculated using the formula 2−ΔΔCt. The primers sequences were showed in the Supplementary Table 1.

*Histology*

Liver specimens were fixed in 4% paraformaldehyde for 2 days, and embedded in paraffin. Sections were cut and subjected to H&E staining, IHC, and immunofluorescence. IHC was used to verify CYLD protein levels and the number of CD3+ cell, CD68+ cell，CD15+ cell, CD56+ cell in the liver of HBV-ACLF patients, CHB patients, and normal controls. Double -labeling immunofluorescence of CD68 and CYLD showed the expression level of CYLD in macrophages. CYLD Mouse mAb and CD3 Rabbit mAb were purchased from Abcam. CD68 Rabbit mAb, CD15 Mouse mAb and CD56 Rabbit mAb were purchased from Cell Signaling Technology.

### *Flow Cytometry*

Immune cell were obtained from Transwell chemotaxis assay. For surface marker staining, cells were labeled with the following mAbs: anti-human CD3 FITC, anti-human CD14 APC, anti-human CD56 PB450, anti-human CD15 PE, anti-human CD19 KO525 (BD Biosciences). After incubation for 20 min at RT, the cells were analyzed using flow cytometer.

***References***

1. Moreau R, Jalan R, Gines P, et al. Acute on chronic liver failure is a distinct syndrome that develops in patients with acute decompensation of cirrhosis. Gastroenterology. 2013;144(7):1426–1437.

2.Terrault NA,  Lok ASF,  McMahon BJ, et al; [Update on prevention, diagnosis, and treatment of chronic hepatitis B: AASLD 2018 hepatitis B guidance.](https://www.geenmedical.com/article?id=29405329&type=true) Hepatology 2018;67(4):1560-1599

1. Wu T, Li J, Shao L, [Xin J](https://www.ncbi.nlm.nih.gov/pubmed/?term=Xin J%5BAuthor%5D&cauthor=true&cauthor_uid=28928275), et al; Chinese group on the Study of Severe Hepatitis B (COSSH). Development of diagnostic criteria and a prognostic score for hepatitis B virus-related acute-on-chronic liver failure. Gut 2018; 67(12): 2181-91.

***Supplementary Figures***

**Figure 1 Biological pathways on ClueGO functional analyses from the overlapping up-regulated DEGs between ACLF vs. CHB and ACLF vs. LC.**


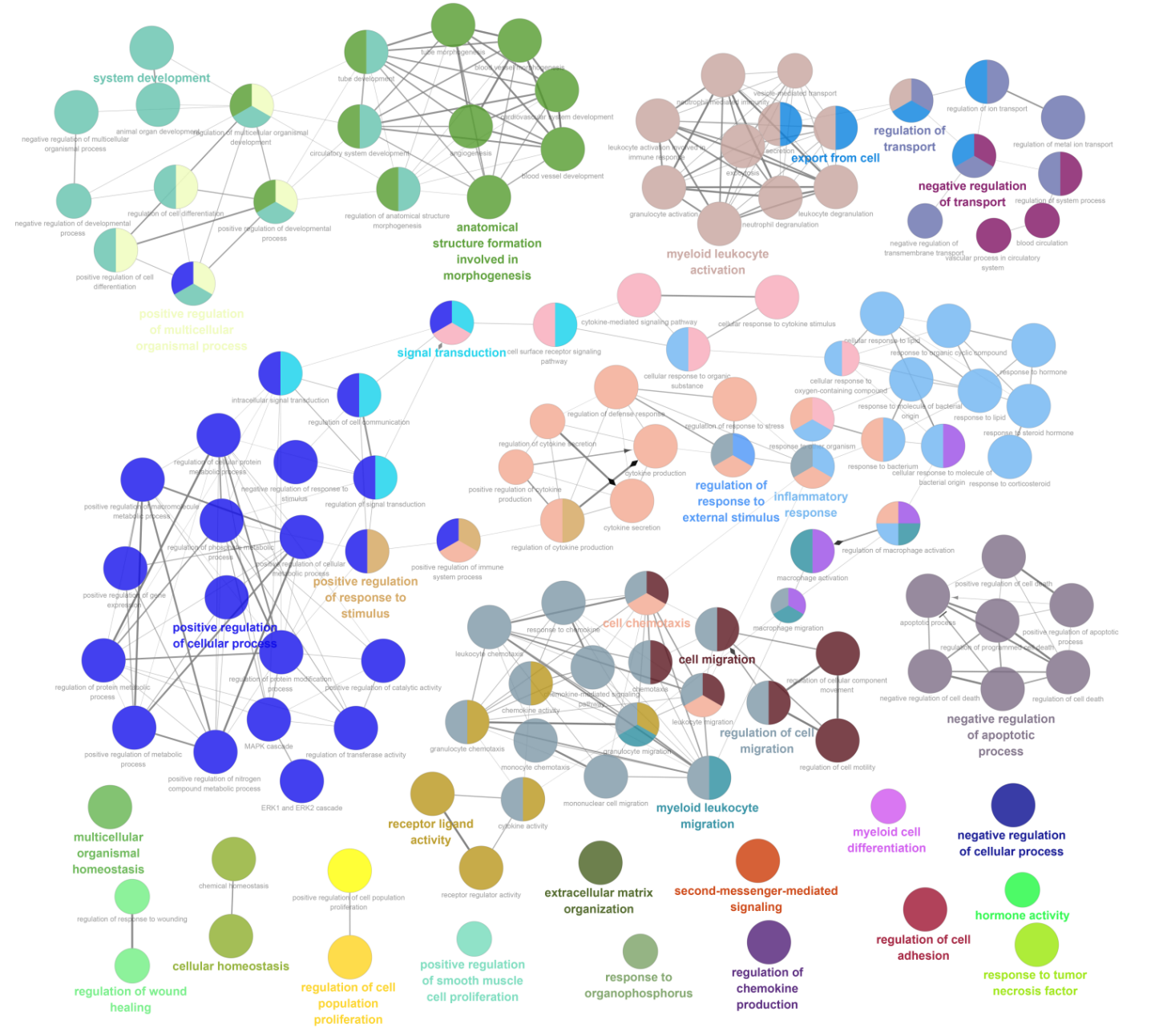


Network of the biological processes identified based on overlapping DEGs.

Abbreviations: differentially expressed genes, DEGs; HBV, hepatitis B virus; ACLF, acute-on-chronic liver failure; CHB, chronic hepatitis B; LC, liver cirrhosis; NC, normal controls.

**Figure 2 ClueGO functional analyses from the overlapping down-regulated DEGs**
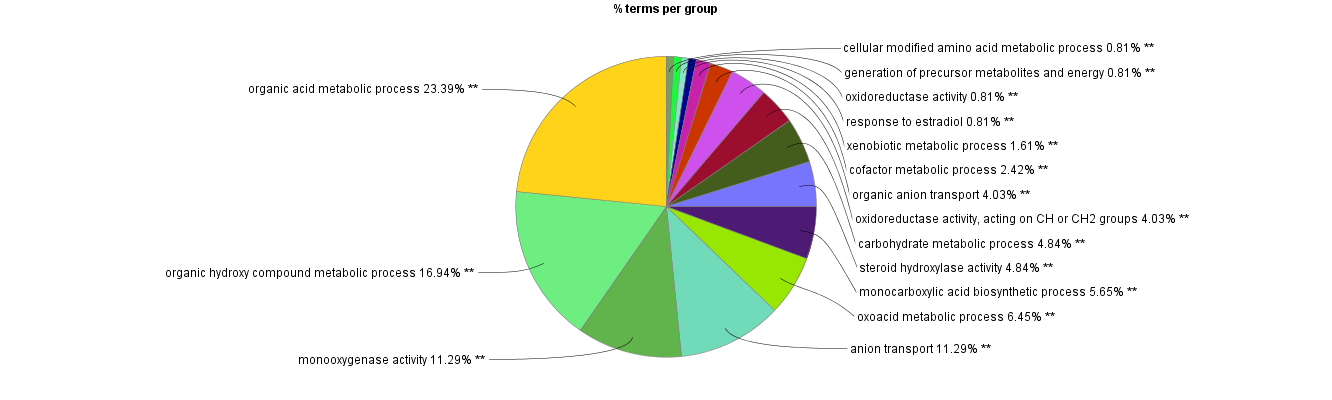


Biological pathways on ClueGO functional analyses from the overlapping down-regulated DEGs of ACLF vs. CHB and ACLF vs. LC.

Abbreviations: differentially expressed genes, DEGs; HBV, hepatitis B virus; ACLF, acute-on-chronic liver failure; CHB, chronic hepatitis B; LC, liver cirrhosis; NC, normal controls.

**Figure 3 The** **comparison of monocyte transcriptomic data between HBV-ACLF and CHB.**

**
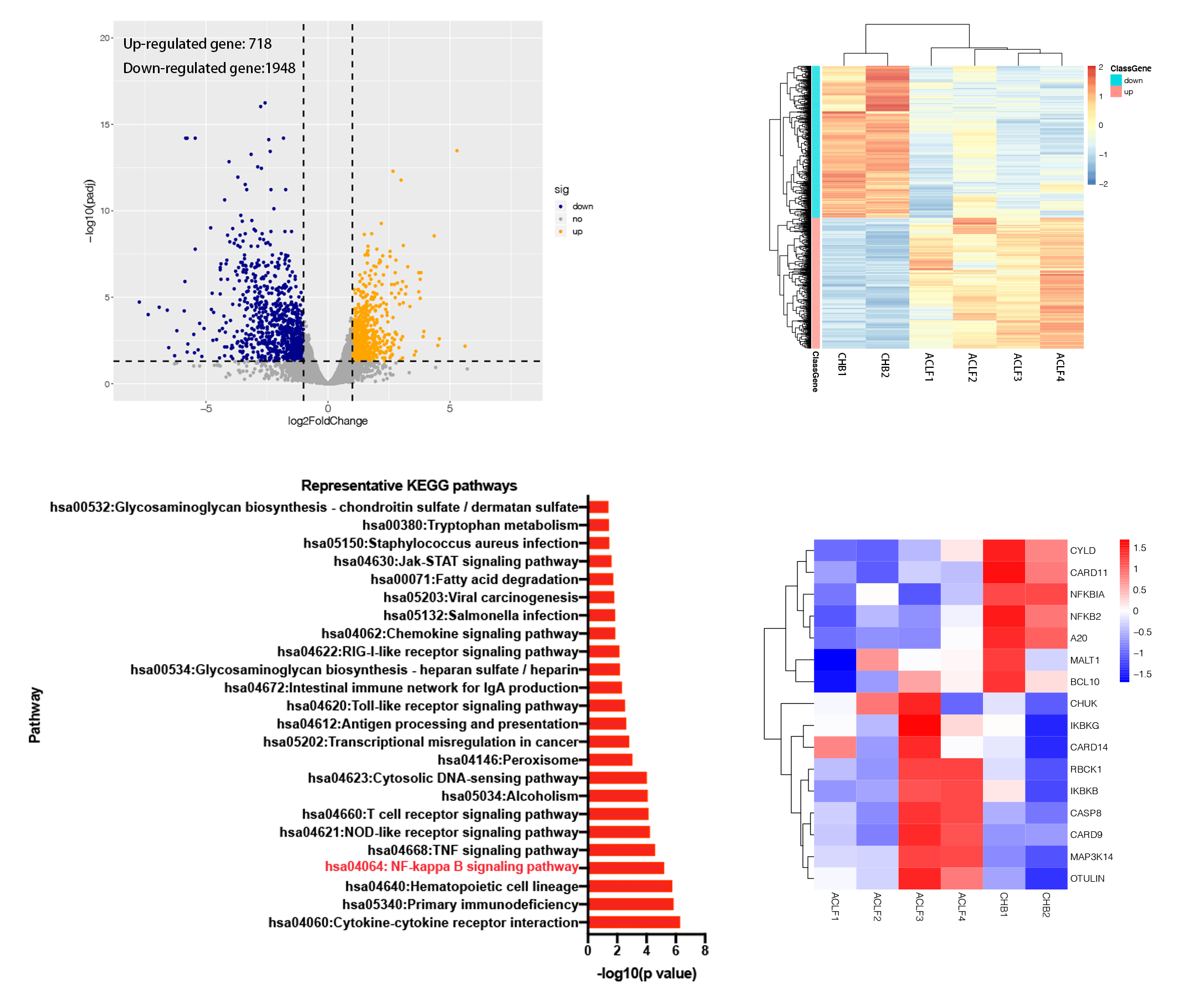
**

Monocytes were sorted from peripheral blood by CD14+ magnetic bead.

1. Volcano plot between ACLF and CHB. (B) Heatmap of DEG in CHB vs. ACLF. (C) KEGG enrichment analysis of DEG in ACLF vs. CHB. (D) Heatmap of genes in the core NF-kB pathway between ACLF and CHB.

Abbreviations: ACLF, acute-on-chronic liver failure; CHB, chronic hepatitis B; KEGG: Kyoto Encyclopedia of Genes and Genomes; DEGs, differentially expressed genes;

**Figure 4. The influence of poly dA:dT and poly I:C on the expression of cytokines and chemokines.**

***
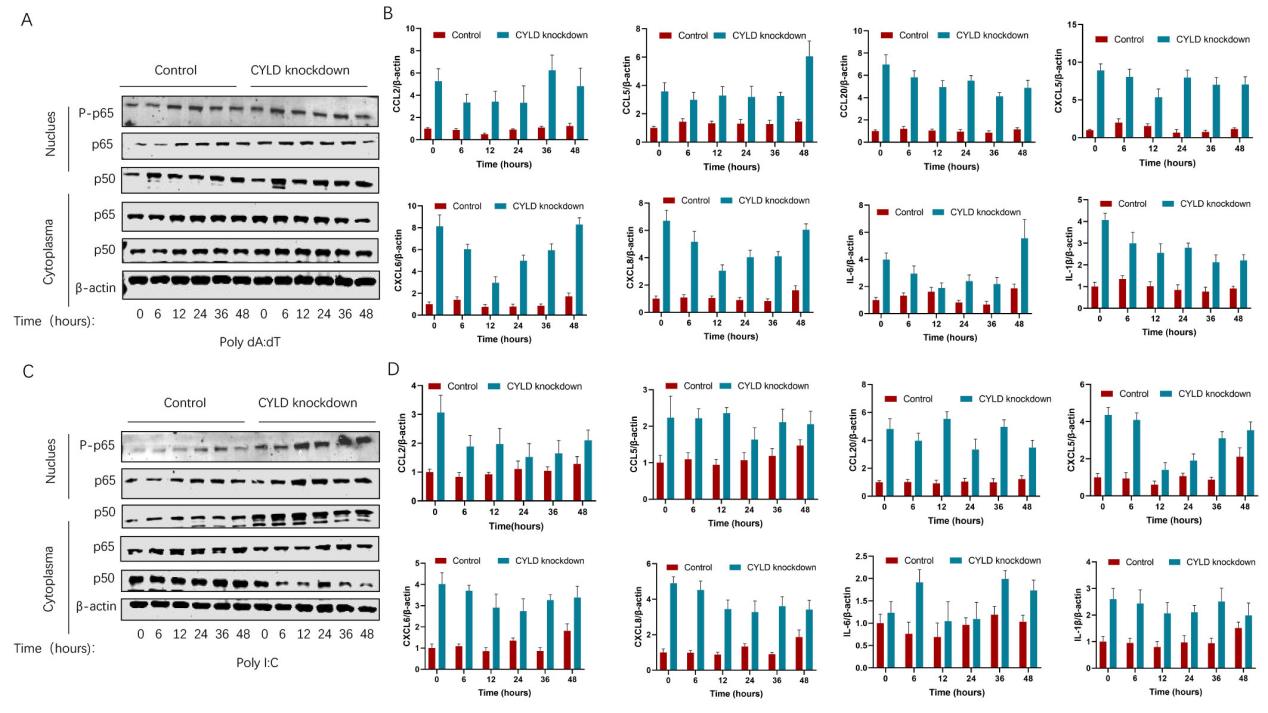
***

THP-1 (8 × 10^5^ cells/ml) were seeded in 6-well cell culture plate added with PMA (100 ng/mL) for 24 h, then were stimulated with poly dA:dT (1 μg/mL) and poly I: C(1 μg/mL) and lysed for collecting cell lysates and supernates at different time point within 48h. The nuclear and cytoplasmic fractions separated from cell lysate to detect protein level of p65,p50 and Phospho-p65. Effects of knockdown CYLD in THP-1 treated with poly dA:dT (1 μg/mL) on NF-ĸB activation (A), proinflammatory cytokine and chemokine production (B). Effects of knockdown CYLD in THP-1 treated with poly I:C (1μg/mL) on NF-ĸB activation (C), proinflammatory cytokine and chemokine production (D).

Values are shown as fold change from control and expressed as the mean, n = 3 (a single sample analyzed in triplicate); bars show SD. *P < 0.05, **P<0.01, ***P < 0.001.

Abbreviations: PMA, Phorbol 12-myristate 13-acetate; CYLD, Cylindromatosis.

**Figure 5 Supernates from CYLD-depleted microphages exhibited an enhanced chemotactic migratory effect**

***
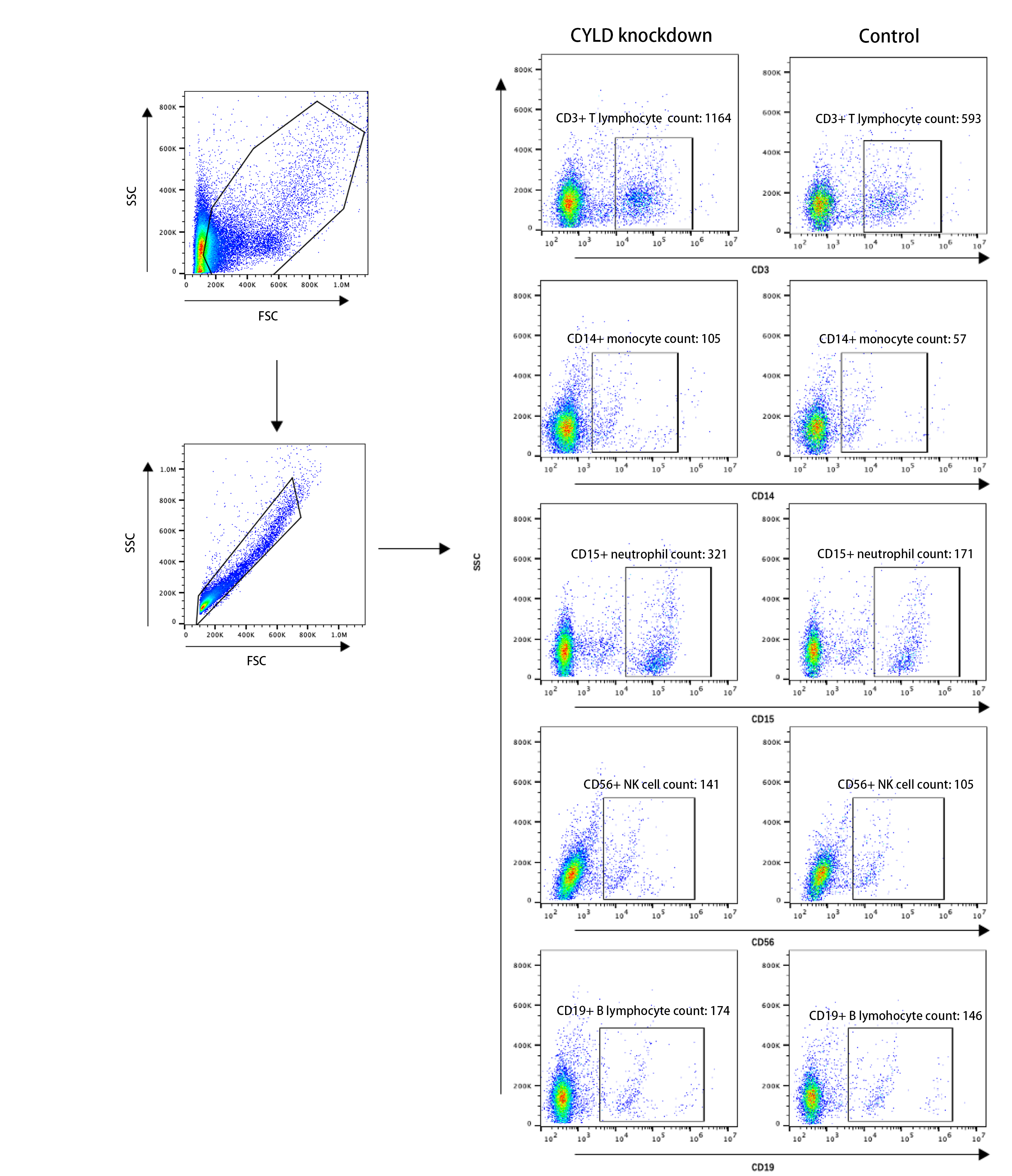
***

Sequential gating strategy for T lymphocyte (CD3+), monocyte (CD14+), neutrophil (CD15+), NK cell (CD56+), B lymphocyte (CD19+) identification using flow cytometry.

***Supplementary Tables***

**Table S1. Clinical characteristics of patients in the RNA-seq group**

|  | **HBV-ACLF (n=6)** | **LC (n = 6)** | **CHB (n=6)** | **NC (n=5)** |
| --- | --- | --- | --- | --- |
| **Clinical data** |  |  |  |  |
| Male sex, % (no.) | 83.3 (5) | 83.3 (5) | 83.3 (5) | 40 (2) |
| Age (yr) | 43 (37 - 55) | 44 (42 - 45) | 47 (43 - 50) | 43 (37 - 55) |
| Hepatic encephalopathy % (no.) | 33.3 (2) | 0 (0) | 0 (0) | 0 (0) |
| Ascites % (no.) | 66.7 (4) | 0 (0) | 0 (0) | 0 (0) |
| **Laboratory data** |  |  |  |  |
| Alanine aminotransferase (U/L) | 340 (255 - 509) | 57.5 (14 - 185) | 29 (20 - 45) | 10 (8 - 13) |
| Albumin (g/L) | 34 (33 - 36) | 44 (42 - 45) | 46 (45 - 49) | 45 (43 - 48) |
| Total bilirubin (µmol/L) | 344.2 (246.9 -464.0) | 15.8 (12.4 - 17.2) | 18.4 (15.1-23.19) | 13.9 ( 12.1-21.2) |
| Creatinine (μmol/L) | 79 (54 - 84) | 70 (65 - 90) | 60 (52 -87) | 52 (45 - 60) |
| White blood cell count (10^9^ /L) | 7.18 (2.3 - 9.84) | 4.89 (4.72 -6.37) | 6.1 (4.84 -6.16) | 5.42 (4.49 - 5.64) |
| Haemoglobin (g/L) | 126 (103 - 126) | 147 (140 - 157) | 145 (139 - 152) | 133 (120 - 149) |
| Platelet count (10^9^ /L) | 90 (43 -114) | 128 (103 - 131) | 149 (139 - 194) | 198 (187- 239) |
| INR | 2.46 (2.21 - 2.67) | 1.09 (0.98 - 1.16) | 1.02 (0.99 - 1.03) | 0.98 (0.95 - 1.05) |
| HBV DNA (log_10_ IU/ml) | 5.24 (3.79 - 5.48) | 4.39 (0-5.88) | 3.65 (0 - 4.42) | - |
| HBsAg (log_10_ IU/ml) | 3.14 (2.86 - 3.20) | 2.79 (2.04-3.05) | 3.38 (3.11 - 3.60) | - |
| HBeAg positive, %(no.) | 33.3 (2) | 50 (3) | 16.7 (1) | - |
| Liver fibrosis, %(no.) |  |  |  |  |
| S0 | - | 0 (0) | 0 (0) | - |
| S1 | - | 0 (0) | 50 (3) | - |
| S2 | - | 0 (0) | 33.3 (2) | - |
| S3 | - | 0 (0) | 16.7 (1) | - |
| S4 | - | 100 (6) | 0 (0) | - |
| liver inflammation, %(no.) |  |  |  |  |
| G0 | - | 0 (0) | 0 (0) | - |
| G1 | - | 50 (3) | 16.7 (1) | - |
| G2 | - | 16.7 (1) | 83.3 (5) | - |
| G3 | - | 33.3 (2) | 0 (0) | - |
| G4 | - | 0 (0) | 0 (0) | - |
| Decompensated cirrhosis, %(no.) | - | 0 (0) | - | - |
| COSSH-ACLF grade, %(no.) |  |  |  |  |
| grade-1 | 50 (3) | - | - | - |
| grade-2 | 50 (3) | - | - | - |
| grade-3 | 0 (0) | - | - | - |

Abbreviations: HBV: hepatitis B virus; ACLF, acute-on-chronic liver failure; LC：liver cirrhosis, INR: international normalized ratio. Data are expressed as the median (interquartile range) or percent (number).

**Table S2. Clinical characteristics of patients in validation group**

|  | **HBV-ACLF (n=24)** | **CHB (n=10)** | **NC (n=14)** |
| --- | --- | --- | --- |
| **Clinical data** |  |  |  |
| Male sex, % (no.) | 95.8 (23) | **80 (8)** | 64.3 (9) |
| Age (yr) | 42 (38 - 47) | 39 (32 - 49) | 40 (33 - 45) |
| Hepatic encephalopathy grade | 29.2 (7) | 0 (0) | 0 (0) |
| Ascites | 66.7 (16) | 0 (0) | 0 (0) |
| **Laboratory data** |  |  |  |
| Alanine aminotransferase (U/L) | 181 (61 -371) | 29 (18 - 35) | 12 (8 - 15) |
| Albumin (g/L) | 36 (33 -38) | 47 (45 - 49) | 46 (44 - 48) |
| Total bilirubin (µmol/L) | 431.6 (323.7 -579.5) | 9.7 (6.7 - 13) | 11.9 (9.1-18.2) |
| Creatinine (μmol/L) | 79 (65 - 87) | 75 (60 - 85) | 55 (40 - 65) |
| White blood cell count (10^9^ /L) | 6.95 (4.61 -8.61) | 6.55 (5.86 - 6.94) | 5.10 (4.12 - 5.64) |
| Haemoglobin (g/L) | 100 (95 -124) | 147 (143 - 158) | 135 (125 - 152) |
| Platelet count (10^9^ /L) | 70 (43 - 99) | 210 (199 - 240) | 200 (178- 254) |
| INR | 2.55 (2.26 - 3.15) | 0.98 (0.94 - 1.02) | 0.99 (0.95 - 1.03) |
| HBV DNA (log_10_ IU/ml) | 5.0 (0 - 5.73) | 6.57 (0 - 8) | - |
| HBsAg (log_10_ IU/ml) | 3.47 (3.00 - 3.85) | 4.23 (3.94 - 4.72) | - |
| HBeAg positive, %(no.) | 29.2 (7) | 60 (6) | - |
| Liver fibrosis, %(no.) |  |  |  |
| S0 | - | 60 (6) | - |
| S1 | - | 20 (2) | - |
| S2 | - | 10 (1) | - |
| S3 | - | 0 (0) | - |
| S4 | - | 10 (1) | - |
| liver inflammation, %(no.) |  |  |  |
| G0 | - | 10 (5) | - |
| G1 | - | 50 (5) | - |
| G2 | - | 40 (4) | - |
| G3 | - | 0 (0) | - |
| G4 | - | 0 (0) | - |
| COSSH-ACLF grade, %(no.) |  |  |  |
| grade-1 | 41.7 (10) | - | - |
| grade-2 | 58.3 (14) | - | - |
| grade-3 | 0 (0) | - | - |

Abbreviations: HBV: hepatitis B virus; ACLF, acute-on-chronic liver failure; LC：liver cirrhosis, INR: international normalized ratio. Data are expressed as the median (interquartile range) or percent (number).

**Table S3. Significantly differential expressed genes**

| Gene Symbol | Ensembl ID | log2Fold Change  (ACLF vs. CHB) | P-value  (ACLF vs. CHB) | log2Fold Change  (ACLF vs. LC) | P-value  (ACLF vs. LC) |
| --- | --- | --- | --- | --- | --- |
| CCL2 | ENSG00000108691 | 4.112345117 | 7.71E-23 | 3.575139026 | 8.73E-18 |
| CCL5 | ENSG00000271503 | 1.20087956 | 5.84E-04 | 1.058732057 | 2.42E-03 |
| CCL20 | ENSG00000115009 | 9.358475496 | 2.76E-20 | 5.411136037 | 8.94E-09 |
| CXCL5 | ENSG00000163735 | 6.213723002 | 5.88E-13 | 3.319962856 | 1.03E-05 |
| CXCL6 | ENSG00000124875 | 5.403679483 | 5.94E-20 | 2.748220337 | 2.33E-06 |
| CXCL8 | ENSG00000169429 | 10.14991632 | 3.10E-25 | 5.620167841 | 2.39E-10 |

P-value computed using DESeq2

**Table S4. Relevant Chemokines in the Pathogenesis of HBV-ACLF and Their Corresponding Receptors and Target cells.**

|  | Alternate name | Main receptor(s) | Target cell(s) |
| --- | --- | --- | --- |
| CCL2 | MCP1 | CCR2 | Monocytes/macrophages, T cell, HSCs |
| CCL5 | RANTES | CCR1, CCR5 | Th1, CD8 T, NK cell, HSCs |
| CCL20 | MIP3α | CCR6 | Th17 |
| CXCL5 | ENA78 | CXCR1 | Neutrophils |
| CXCL6 | GCP2 | CXCR1, CXCR2 | Neutrophils, monocytes |
| CXCL8 | IL-8 | CXCR1, CXCR2 | Neutrophils, monocytes |

Abbreviations: hepatitis B virus, HBV; ACLF, acute-on-chronic liver failure; Treg: regulatory T cell; [Hepatic Stellate Cells](http://www.sohu.com/a/241703279_819877),HSCs; natural killer cell, NK cell.
